# *Plasmodium knowlesi*: Reservoir Hosts and Tracking the Emergence in Humans and Macaques

**DOI:** 10.1371/journal.ppat.1002015

**Published:** 2011-04-07

**Authors:** Kim-Sung Lee, Paul C. S. Divis, Siti Khatijah Zakaria, Asmad Matusop, Roynston A. Julin, David J. Conway, Janet Cox-Singh, Balbir Singh

**Affiliations:** 1 Malaria Research Centre, Faculty of Medicine and Health Sciences, University Malaysia Sarawak, Kuching, Sarawak, Malaysia; 2 Sarawak State Health Department, Kuching, Sarawak, Malaysia; 3 Department of Pathogen Molecular Biology, London School of Hygiene and Tropical Medicine, London, United Kingdom; 4 Centre for Infection, St. George's University of London, London, United Kingdom; Case Western Reserve University, United States of America

## Abstract

*Plasmodium knowlesi*, a malaria parasite originally thought to be restricted to macaques in Southeast Asia, has recently been recognized as a significant cause of human malaria. Unlike the benign and morphologically similar *P. malariae*, these parasites can lead to fatal infections. Malaria parasites, including *P. knowlesi*, have not yet been detected in macaques of the Kapit Division of Malaysian Borneo, where the majority of human knowlesi malaria cases have been reported. In order to extend our understanding of the epidemiology and evolutionary history of *P. knowlesi*, we examined 108 wild macaques for malaria parasites and sequenced the circumsporozoite protein (*csp*) gene and mitochondrial (mt) DNA of *P. knowlesi* isolates derived from macaques and humans. We detected five species of *Plasmodium* (*P. knowlesi*, *P. inui*, *P. cynomolgi*, *P. fieldi* and *P. coatneyi*) in the long-tailed and pig-tailed macaques, and an extremely high prevalence of *P. inui* and *P. knowlesi*. Macaques had a higher number of *P. knowlesi* genotypes per infection than humans, and some diverse alleles of the *P. knowlesi csp* gene and certain mtDNA haplotypes were shared between both hosts. Analyses of DNA sequence data indicate that there are no mtDNA lineages associated exclusively with either host. Furthermore, our analyses of the mtDNA data reveal that *P. knowlesi* is derived from an ancestral parasite population that existed prior to human settlement in Southeast Asia, and underwent significant population expansion approximately 30,000–40,000 years ago. Our results indicate that human infections with *P. knowlesi* are not newly emergent in Southeast Asia and that knowlesi malaria is primarily a zoonosis with wild macaques as the reservoir hosts. However, ongoing ecological changes resulting from deforestation, with an associated increase in the human population, could enable this pathogenic species of *Plasmodium* to switch to humans as the preferred host.

## Author Summary

We recently described the first focus of human infections with *P. knowlesi*, a malaria parasite of monkeys, and subsequently reported that these infections can be fatal. Whether mosquito transmission of infection depended on the monkey reservoir or was maintained by the human population was unknown. In the area of highest human infection incidence (within the Kapit Division of Sarawak, Malaysian Borneo), we surveyed 108 wild monkeys and found most were infected with malaria parasites, including *P. knowlesi*. We observed that the number of *P. knowlesi* genotypes per infection was much higher in monkeys than humans, some genotypes were shared between the two hosts and no major types were associated exclusively with either host. Evolutionary analyses of sequence data indicate that *P. knowlesi* existed in monkeys prior to human settlement in Southeast Asia and underwent a recent population expansion. Thus, *P. knowlesi* is essentially zoonotic; humans being infected with these parasites from the original and reservoir monkey hosts probably since they first entered the forests of Southeast Asia. We consider that the current increase in the human population, coupled with ecological changes due to deforestation, could result in a switch to humans as the preferred host for this pathogenic *Plasmodium* species.

## Introduction

Until recently, it was believed that malaria in humans was caused by only four species of parasite (*Plasmodium falciparum*, *P. vivax*, *P. malariae* and *P. ovale*). However, this perception changed when we discovered a large focus of human infections with *P. knowlesi* in the Kapit Division of Sarawak, Malaysian Borneo [1]. These infections had predominantly been mistakenly identified as *P. malariae* by microscopy, since both species have similar morphological characteristics [1], [2]. With subsequent reports of human infections in other parts of Malaysia [3], [4], and in Thailand [5], [6], Myanmar [7], Singapore [8], [9], the Philippines [10], Vietnam [11] and Indonesia [12], [13], *P. knowlesi* is now recognized as the fifth species of *Plasmodium* responsible for human malaria. It causes a wide spectrum of disease and can lead to high parasite counts, severe complications and death [3], [14]. In a recent study, we found that approximately 1 in 10 knowlesi malaria patients at Kapit Hospital developed potentially fatal complications, comparable to *P. falciparum* malaria, which is considered to be the most virulent type of malaria in humans [14].

*P. knowlesi* is primarily a simian malaria parasite and was first isolated from a long- tailed macaque (*Macaca fascicularis*) imported to India from Singapore in 1931 [15]. Subsequently, *P. knowlesi* has been detected in wild long-tailed macaques of Peninsular Malaysia [4], [16] and the Philippines [17], in pig-tailed macaques (*M. nemestrina*) of Peninsular Malaysia [16] and in banded leaf monkeys (*Presbytis melalophus*) in Peninsular Malaysia [16]. There has been no documented evidence of *P. knowlesi* or any other malaria parasites in monkeys in Malaysian Borneo, and although a monkey source for the hundreds of human *P. knowlesi* infections that have been described in the Kapit Division of Sarawak [1], [3], [14] appeared likely, it remained to be proven.

Prior to our report in 2004 of the large focus of human infections in Sarawak, Malaysian Borneo, when we utilized molecular methods for characterisation and PCR assays for detection of *P. knowlesi* [1], there had been only one confirmed case of a naturally-acquired *P. knowlesi* infection in a human [18]. That person got infected with *P. knowlesi* while spending a few weeks in the forest of Pahang, Peninsular Malaysia in 1965. It is not known whether the large focus in Malaysian Borneo and subsequent recent reports of human knowlesi malaria in Southeast Asia represent a truly newly emergent malaria parasite in humans or whether human infections have been occurring for a relatively long period, but have gone undetected due to unavailability of molecular detection methods to distinguish between *P. knowlesi* and *P. malariae*. In order to identify the reservoir hosts and extend our understanding of the epidemiology and evolutionary history of *P. knowlesi*, we examined blood samples from wild macaques for malaria parasites, and analyzed the circumsporozoite protein (*csp*) gene and the mitochondrial (mt) genome of *P. knowlesi* isolates derived from humans and macaques.

## Results

Nested PCR examination of blood samples from 108 wild macaques (82 long-tailed, 26 pig-tailed), sampled from 17 different locations in the Kapit Division of Sarawak, showed that 101 (94%) of the macaques were infected with malaria parasites. Long-tailed macaques had a higher prevalence of infection (98%) than pig-tailed macaques (81%) (Fisher's Exact P = 0.009) (Table 1). By nested PCR assays, we detected 5 species of *Plasmodium*, with *P. inui* being the most common (prevalence of 82%), followed by *P. knowlesi* (78%), *P. coatneyi* (66%), *P. cynomolgi* (56%), and *P. fieldi* (4%). Multiple species infections were very common, with 91 of the 108 (84%) macaques being infected by two or more species of *Plasmodium* each. There was a higher prevalence of *P. knowlesi* among long-tailed macaques (87%) than pig-tailed macaques (50%) (P = 0.006).

**Table 1:** Summary of malaria parasite infections in wild macaques.

		No. of macaques infected		
Infection	*Plasmodium* spp.	LT	PT	Total
Single	Pk		1	1
	Pct	3		3
	Pcy	1		1
	Pin	2	3	5
Double	Pk, Pct	1		1
	Pk, Pcy	2		2
	Pk, Pfi	1		1
	Pk, Pin	5	3	8
	Pcy, Pin	2	2	4
	Pin, Pct		2	2
Triple	Pk, Pcy, Pct	3		3
	Pk, Pcy, Pin	4	3	7
	Pk, Pin, Pct	14	3	17
	Pk, Pin, Pfi	1		1
	Pcy, Pin, Pct	1	1	2
Quadruple	Pk, Pcy, Pin, Pct	38	3	41
	Pk, Pin, Pct, Pfi	1		1
Quintuple	Pk, Pcy, Pin, Pct, Pfi	1		1
Total *Plasmodium*-positive		80	21	101
Total *Plasmodium*-negative		2	5	7
Total no. of macaques		82	26	108

Pk = *P. knowlesi*, Pct = *P. coatneyi*, Pcy = *P. cynomolgi*, Pin = *P. inui*, Pfi = *P. fieldi*. LT = Long-tailed, PT = Pig-tailed.

**Figure 1:**
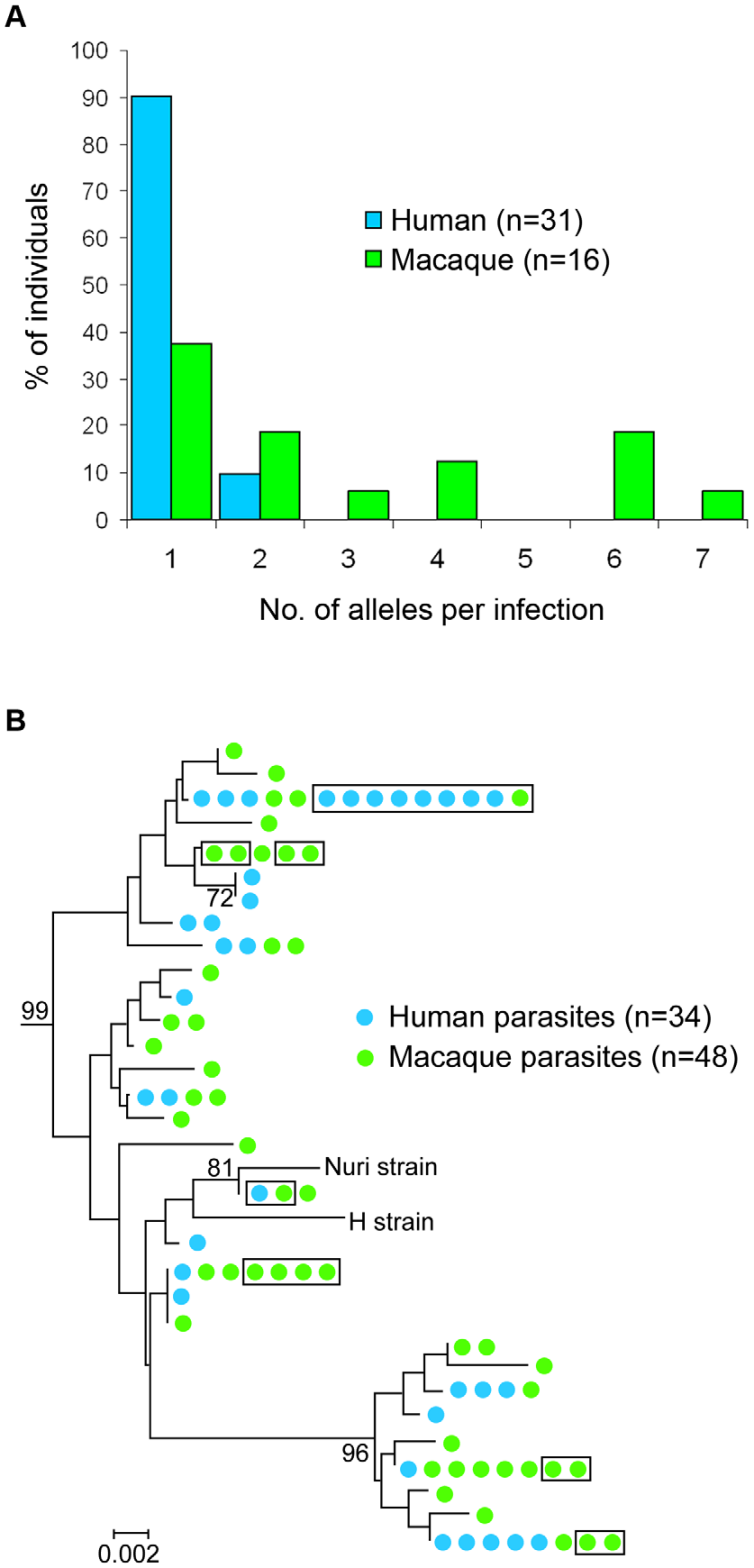
Analyses of *P. knowlesi csp* gene sequences from infections of macaques and humans. (**A**) Histogram showing proportion of human and macaque individuals with different numbers of full length *csp* alleles detected per infection. (**B**) Diversity of *csp* alleles in the *P. knowlesi* clade of the phylogenetic tree of *Plasmodium* spp. (Fig. S1), based on the non-repeat region of the gene. These intraspecific relationships clustered by the neighbor-joining method on a Kimura 2-parameter distance matrix represent observed pairwise sequence similarity (phylogeny cannot be determined within the species for a nuclear gene due to recombination). Figures on the branches are bootstrap percentages based on 1,000 replicates and only those above 70% are shown. The horizontal branch lengths indicate nucleotide differences per site compared with the scale bar. Parasite clones in the boxes represent sequences that are completely identical for the whole *csp* gene (including repeat sequences not analysed by alignment but given separately in Supplementary Table S1).

To compare the molecular identity of the parasites in macaques and humans, we first sequenced the *P. knowlesi csp* gene in blood samples from 31 patients admitted to Kapit Hospital and 16 wild macaques. Most macaques (10 of 16), but only a minority of humans (3 of 31) contained 2 or more *csp* alleles (Fig. 1A). Overall, we derived 48 *csp* allele sequences of *P. knowlesi* from the macaques and 34 from the human samples, with 61 different alleles observed in total. Three of these *csp* alleles were shared between human and macaques, three were shared by macaques, and the remaining alleles were detected in only macaques or humans (Fig. S1 and Fig. 1B). We found that the central region of the *P. knowlesi csp* was composed of highly polymorphic repeat sequences (Table S1). Analysis of the aligned non-repeat regions of *csp* showed 19 polymorphic sites, of which 14 were shared polymorphisms in samples from both host populations (Fig. S2). The nucleotide diversity of *csp* was similar in both hosts (π = 2.2×10^−2^ in humans and 2.4×10^−2^ in macaques), although the haplotype diversity was marginally higher in macaques (H = 0.82, SD = 0.03) than in humans (H = 0.73, SD = 0.06). There was no clustering of *csp* allele sequence type associated with either host (Fig. 1B).

**Figure 2:**
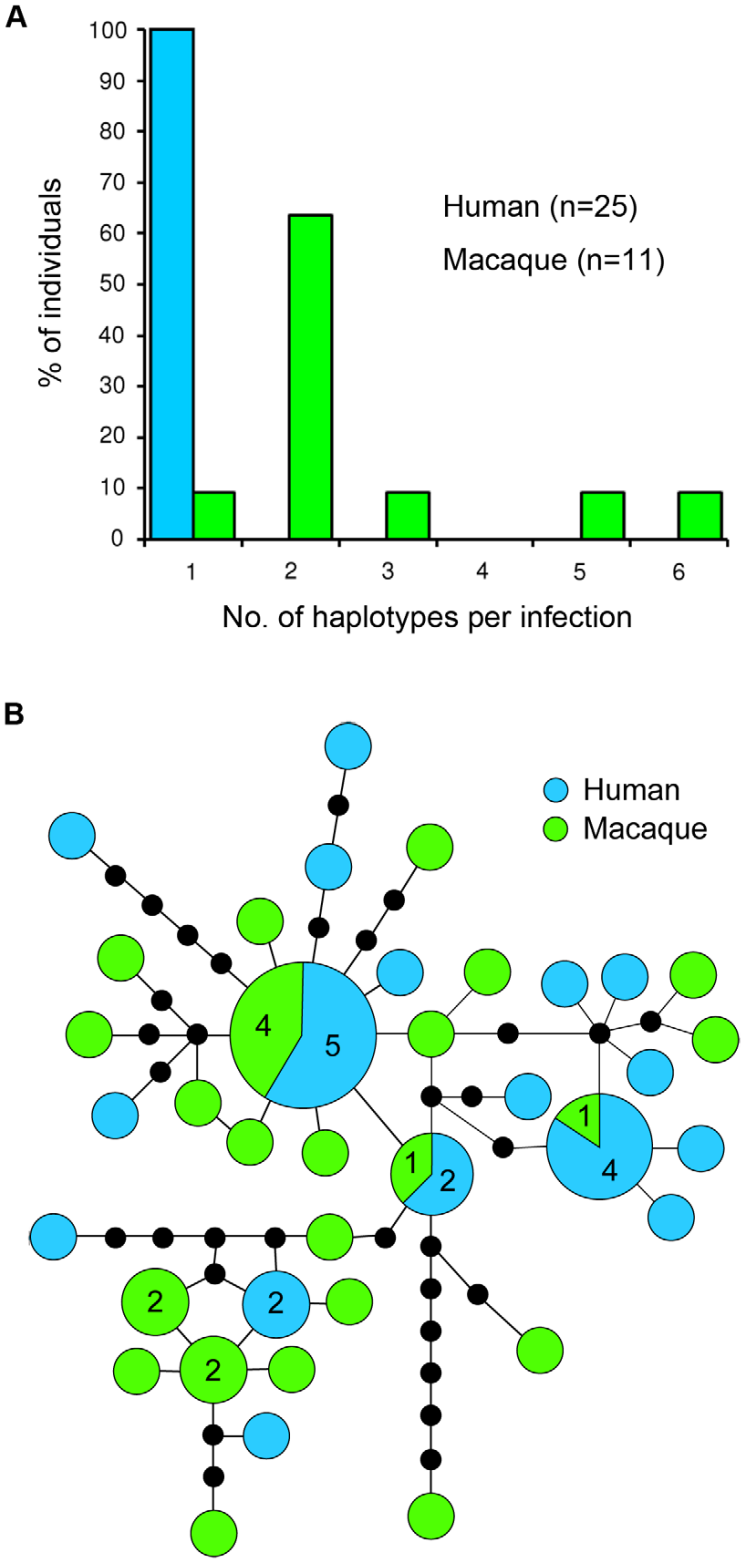
Diversity and haplotype network of *P. knowlesi* mtDNA genome. (**A**) Histogram showing proportion of human and macaque individuals with different numbers of mtDNA haplotypes detected per infection. (**B**) Schematic diagram of genealogical network showing relationship among 37 mtDNA haplotypes of *P. knowlesi*. Numbers in larger circles represent number of haplotypes and unnumbered circles represent a single haplotype. Each line connecting the circles represents a mutational step and black dots represent hypothetical missing intermediates.

We also sequenced the ∼6-kilobase mtDNA genome of *P. knowlesi* parasites isolated from 25 malaria patients and 11 macaques. Each human sample had a single mtDNA haplotype, while all except one macaque sample contained multiple (2 to 6) haplotypes (Fig. 2A). In total, we generated 54 complete mtDNA genome sequences, representing 37 different mtDNA haplotypes, with a higher number of haplotypes in the macaques (23 haplotypes from 11 samples) than in the humans (17 haplotypes from 25 samples). Six of the haplotypes were found in more than 1 sample, and 3 of these were shared between the human and macaque hosts (Fig. 2B). Forty-five single nucleotide polymorphisms (SNPs) and a 4-base insertion/deletion within the *P. knowlesi* mtDNA genome were identified (Fig. S3), and the level of nucleotide diversity (π) of mtDNA was estimated as 7.5±0.7×10^−4^.

**Figure 3:**
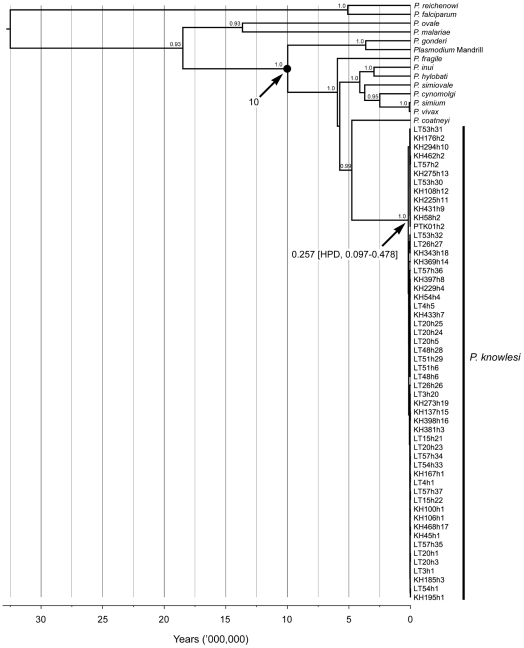
Time-calibrated maximum clade credibility phylogeny based on the 6 kb mtDNA of Plasmodium species of human and non-human primates. Phylogenetic tree scaled to time generated using uncorrelated relaxed clock model and Bayesian skyline coalescent tree prior, with the divergence of *Plasmodium* spp. of Asian macaques and *P. gonderi/Plasmodium* sp. (Mandrill) as the calibration point (black circle). TMRCAs and HPDs for P. knowlesi and *Plasmodium* of Asian macaques are indicated. Numbers on branches are values of posterior probabilities. The accession numbers of sequence data of P. knowlesi were deposited in GenBank under the accession numbers EU880446–EU880499 and accession numbers of the other sequences are provided in the Methods section.

The Bayesian coalescent approach [19] was used to estimate the time to the most recent common ancestor (TMRCA) for *P. knowlesi*. A nucleotide substitution rate for the mtDNA genome of 3.13×10^−9^ (95% HPD, 1.94–4.45×10^−9^) substitutions per site per year was estimated by comparing mtDNA sequences of *P. knowlesi*, *P. fragile*, *P. cynomolgi*, *P. simiovale* (parasites of Asian macaques) with *P. gonderi* (a parasite of African mangabeys) and *Plasmodium* sp. (Mandrill), assuming parasite lineages separated when Asian Old World monkeys and African Old World monkeys diverged 10 million years ago (MYA) [20]. This derived rate yielded an estimate of 257,000 (95% HPD: 98,000–478,000) years before present as the TMRCA of *P. knowlesi* (Fig. 3).

There was no evidence of recombination in the mtDNA of *P. knowlesi* (Table S2), and reconstruction of the haplotype genealogical network demonstrated that no distinct lineages of mtDNA were exclusively associated with either human or macaque hosts (Fig. 2B). Our phylogeny-trait association tests based on association index (AI) [21] and parsimony score (PS) [22] of host-parasite phylogenetic substructure do not reject the null hypothesis of no association between parasite and host (Table S3). Hence, this further indicates the absence of a distinct lineage of *P. knowlesi* parasites associated with either human or macaque hosts.

**Figure 4:**
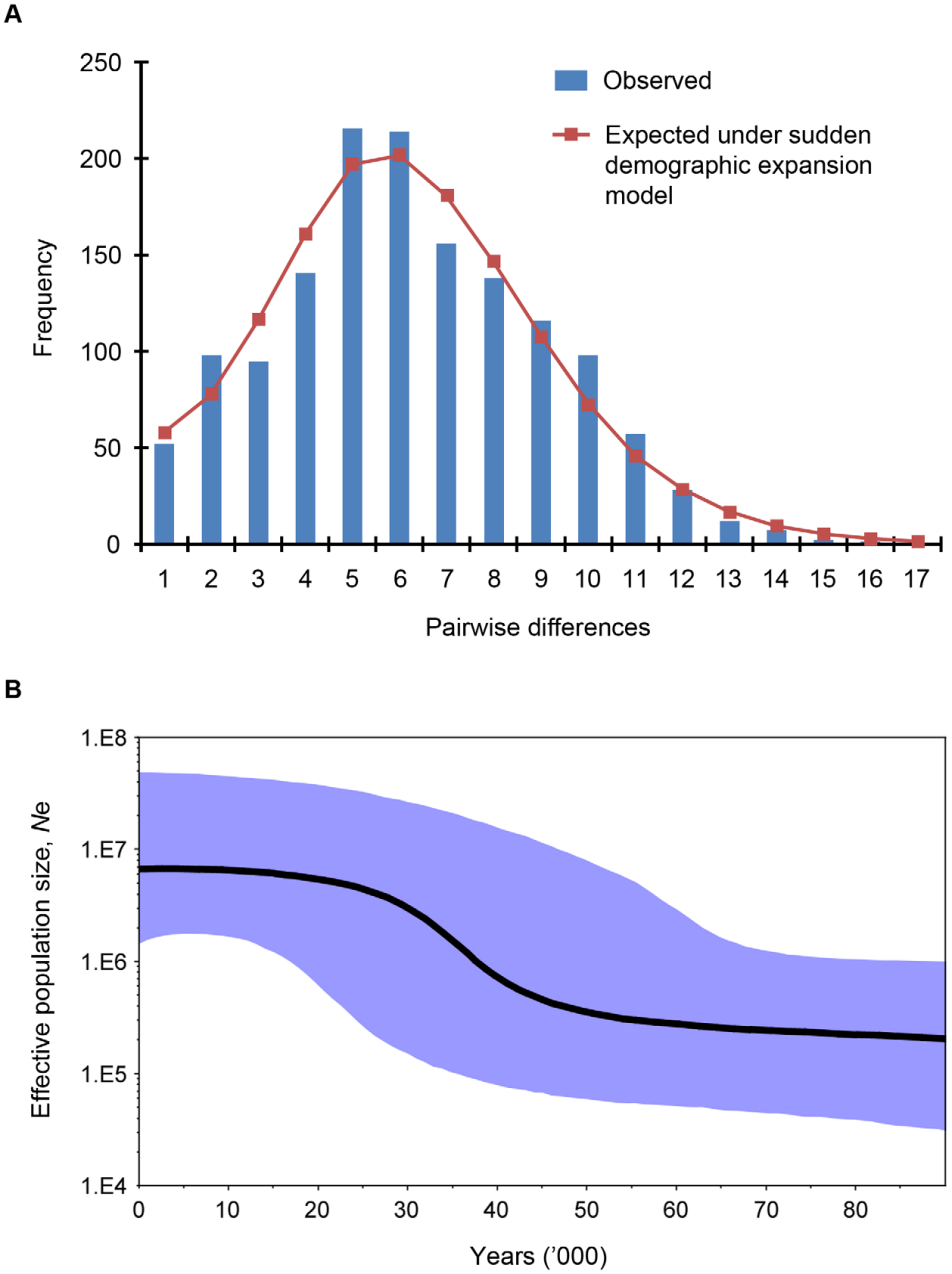
Demographic and evolutionary history of *P. knowlesi*. (**A**) Pairwise mismatch distribution of the *P. knowlesi* mt genome. The bars represent observed frequency of the pairwise differences among mtDNA sequences and the line represents the expected curve for a population that has undergone a demographic expansion. (**B**) Bayesian skyline plot showing changes in effective population size (*Ne*) through time as estimated using uncorrelated log-normal relaxed molecular clock and Bayesian skyline coalescent model (10 coalescent-interval groups) with the substitution rate of 3.13×10^−9^ substitutions per site per year. The y-axis representing the effective population size is given on a logarithmic scale and the x-axis represents time in thousands of years ago. The thick solid black line is the median estimate and the blue shaded area represents the 95% highest probability density (HPD) intervals for effective population size.

We observed an excess of unique mtDNA haplotypes, which appear at the edges of a star-like structure of the haplotype genealogical network (Fig. 2B), and this is indicative of an evolutionarily recent population expansion of *P. knowlesi*. The signature of population expansion is also evident from the unimodal shape of the pairwise mismatch distribution (Fig. 4A), and this is supported by a low Harpending's raggedness index (*r* = 0.009, *P* = 0.87) [23]. In addition we used the Tajima's D [24], Fu and Li's D and F (with out-group) [25] and Fay and Wu's H [26] statistics to detect deviation from the model of neutral evolution considering that the deviation from neutrality could be due to demographic processes such as population expansion, population bottleneck or mutation rate heterogeneity [27]. We obtained significant negative values for all these statistics (Tajima's D = −1.88, *P* = 0.001; Fu and Li's D = −2.60, P = 0.02; Fu and Li's F = −2.80, P = 0.009; Fay and Wu's H = −10.33, P = 0.044), thereby providing further evidence for an expansion of the *P. knowlesi* parasite population.

To further investigate the demographic history of *P. knowlesi*, we estimated the changes in effective population size of the parasite through time, using a coalescent approach called the Bayesian skyline plot [19]. The plot indicates that *P. knowlesi* underwent a rapid population growth between approximately 30,000 and 40,000 years before present (Fig. 4B). In addition, we performed independent analyses for *P. knowlesi* mtDNA sequences derived from humans and macaques. Similar trends were reflected in the Bayesian skyline plots for each host (Fig. S4), showing that there are no differences between the demographic history of *P. knowlesi* for either host.

We analysed mtDNA cytochrome b sequence data for long-tailed macaques in Southeast Asia using a similar approach, but did not find any evidence for changes in population size between 100,000 and 10,000 years before present (Fig. S5).

## Discussion

Our study shows that wild macaques in the Kapit Division of Sarawak, Malaysian Borneo are infected with the same 5 species of *Plasmodium* found in macaques of Peninsular Malaysia [16], [28], and that these macaques have a very high prevalence of *P. knowlesi* and *P. inui*. In previous studies, we found that *P. knowlesi* is the most common cause of hospital admission for malaria in the Kapit Division and there are approximately 90 knowlesi malaria admissions, predominantly adults, at Kapit Hospital per year [1], [3], [14]. The actual annual incidence of knowlesi malaria for the Kapit Division is probably higher, because not all persons with *P. knowlesi* infections may have sought treatment in hospital and there may be asymptomatic infections and misdiagnoses. Nevertheless, the restricted number of knowlesi malaria cases in the human population of 109,000 [14], contrasts with the extremely high prevalence of *P. knowlesi* we detected in the wild macaques of the Kapit Division. These findings contrast with the absence of *P. knowlesi* infections in a survey of 99 long-tailed macaques in one region in Thailand [29]. In that study, the majority of macaques were trapped near a temple in a region where very few human knowlesi malaria cases have been reported [6], and the absence of detectable *P. knowlesi* there could be due to the low abundance of mosquitoes of the *Anopheles leucosphyrus* group, which have been shown to be the most competent vectors of knowlesi malaria [28]. We previously identified one member of this group, *Anopheles latens*, as the vector for *P. knowlesi* in the Kapit Division [30]. This mosquito feeds outdoors after dusk and is attracted to humans and macaques at ground level, but prefers to feed on macaques at a higher elevation [31]. Our findings here, of the higher number of *P. knowlesi csp* alleles and mtDNA genome haplotypes detected per infection in macaques compared with humans, and the very high prevalence of *P. knowlesi* in macaques, suggest that presently there is a greater intensity of transmission of *P. knowlesi* by the vectors among wild macaques, than from macaques to humans. These results, including our observation that certain alleles of the *P. knowlesi csp* gene and mtDNA genome haplotypes are shared between macaque and human hosts, taken together with previous epidemiological [1], [3], [14] and entomological data [30], [31], strongly indicate that knowlesi malaria is a zoonosis in the Kapit Division and that wild macaques are the reservoir hosts.

Our estimated TMRCA for *P. knowlesi* (98,000–478,000 years ago) indicates that *P. knowlesi* is derived from an ancestral parasite population that predates human settlement in Southeast Asia [32], [33]. Therefore macaques, which colonized Asia more than 5 million years ago [34], were the most likely hosts during the initial emergence of *P. knowlesi* in this region. Our estimate also indicates that *P. knowlesi* is as old as, or older than the 2 most common human malaria parasites, *P. falciparum* and *P. vivax*, for which the TMRCA has been estimated to be 50,000–330,000 [35], [36] years and 53,000–265,000 years [37], [38], respectively.

Our analyses of the mtDNA data indicate that that *P. knowlesi* underwent a period of population expansion, estimated at 30,000–40,000 years ago, which coincides with a time when Borneo was part of mainland Southeast Asia [39] and the possibility of increased parasite admixture between macaque troops. This period is concordant with a time of exceptional human population growth in Southeast Asia, based on mtDNA sequence analysis [40]. We did not detect a similar population expansion of macaques, but this analysis was based on the cytochrome b gene alone. It would be preferable to analyze mtDNA sequences of macaques sampled in Borneo to determine whether they underwent a parallel historical population expansion. It is possible that the population expansion of *P. knowlesi* was not directly linked to expansion in any primate host, but was rather due to the expansion or adaptation of the mosquito vectors.

In conclusion, our results indicate that *P. knowlesi* in Sarawak is zoonotic, with humans sharing parasites with the original and preferred hosts, the macaques, most likely since they first came into close contact in the forests of Southeast Asia. A multi-gene family (*KIR*) in *P. knowlesi* encodes proteins with sequence motifs mimicking host cell receptor CD99 in macaques [41], and the observation that the *KIR* motifs are less perfectly matched to the human CD99 sequence also supports the hypothesis that the parasite is particularly adapted to macaque hosts. Humans acquire knowlesi malaria on occasions when they enter the habitats shared by macaques and mosquitoes of the *Anopheles leucosphyrus* group [4], [16], [28], [30], which are forest-dwelling mosquitoes that feed outdoors after dusk [28], [31]. There is no evidence yet to suggest a host-switch by *P. knowlesi*, unlike other human malaria parasites such as *P. vivax* and *P. falciparum* that might have been part of ancient zoonoses [38], [42], but have since adapted to humans. However, it is possible that the current destruction of the natural forest ecosystem, with associated increase of the human population, may alter the parasite, macaque host and mosquito population dynamics and lead to an adaptive host-switch of *P. knowlesi* to humans.

## Materials and Methods

### Ethics statement

Currently, Malaysia has no legislation governing the use of animals in research. Nevertheless, this study was carried out in strict accordance with the recommendations by the Sarawak Forestry Department for the capture, use and release of wild macaques. A veterinarian took blood samples from macaques following anesthesia by intramuscular injection of tiletamine and zolazepam. All efforts were made to minimize suffering by collecting blood from macaques at the trap sites and releasing the animals immediately after the blood samples had been obtained. The Sarawak Forestry Department approved the study protocol for capture, collection of blood samples and release of wild macaques (Permits Numbers: NPW.907.4.2-32, NPW.907.4.2-97, NPW.907.4.2-98, 57/2006 and 70/2007). A permit to access and collect macaque blood samples for the purpose of research was also obtained from the Sarawak Biodiversity Centre (Permit Number: SBC-RP-0081-BS). Human blood samples were taken after written informed consent had been obtained from patients admitted to Kapit Hospital. This study was approved by the Medical Research and Ethics Committee of the Malaysian Ministry of Health (Reference number: KKM/JEPP/02 Jld.2 [133]), which operates in accordance to the International Conference of Harmonization Good Clinical Practice Guidelines.

### Samples from macaques and humans

A total of 108 macaques were sampled from 2004 to 2008. Ninety were from 5 major sites and the remainder from 12 different locations in the Kapit Division of Sarawak. All locations were within 2 km from longhouse communities where human knowlesi cases had previously been reported. After blood was obtained from anaesthetised animals, they were tagged with a microchip (to prevent re-sampling) and released. Human blood samples were obtained from 31 patients with knowlesi malaria admitted to Kapit Hospital between 2000 and 2006.

### Nested PCR assays

DNA was extracted from macaque and human blood samples as described previously [1]. DNA samples from macaques were examined using nested PCR assays with genus and species-specific primers based on the small subunit ribosomal RNA genes [1]. PCR primer sequences (for *P. knowlesi*, *P. coatneyi*, *P. cynomolgi*, *P. fieldi* and *P. inui*) and annealing temperatures are provided in Table S4.

### Sequencing of *csp* and mtDNA

We amplified and sequenced the complete *P. knowlesi csp* gene and performed sequence analysis as described previously [1]. At least two clones from each of the two PCR amplifications per sample were sequenced and for macaque samples, at times 5 PCR amplifications and cloning procedures were necessary before *P. knowlesi* sequences could be obtained. For amplification of the *P. knowlesi* mtDNA, two back-to-back primers were designed (Pkmt-F1, 5′- GGACTTCCTGACGTTTAATAACGAT-3′ and Pkmt-R1, 5′-TGGACGTTGAATCCAATAGCGTA-3′) by using previously described mtDNA sequence of *P. knowlesi* [43]. PCR amplification was performed separately for each sample to prevent cross-contamination of DNA using the Elongase Amplification System (Invitrogen). The PCR product for each isolate was gel purified, cloned into pCR-XL-TOPO vector (Invitrogen) and sequenced using BigDye Terminator Cycle Sequencing kit (Applied Biosystems) with 28 internal primers (sequences in Table S5) that enabled sequencing of both DNA strands.

The diversity of *P. knowlesi* mtDNA from 6 human samples was initially characterized. These were selected based on the criteria that each patient originated from a different geographical area of Kapit Division and patients had no records of recent travel history. Only females were chosen assuming that females travel less than the males. Sequencing data was obtained from at least 2 clones originating from separate PCR amplifications. Both DNA strands were sequenced from each clone and any nucleotide conflicts found were resolved following a third PCR amplification, cloning and sequencing.

The remaining 19 human samples were randomly chosen from patients admitted between 2000 and 2006. Following PCR amplification and cloning, these samples were haplotyped by sequencing single DNA strand of the mt genome and at least 2 plasmid clones were sequenced for each sample. Any single nucleotide polymorphisms (SNPs) or singleton polymorphisms detected were verified by sequencing the polymorphic regions in at least 2 plasmid clones originating from separate PCR amplifications, and both DNA strands were sequenced.

### Sequence analysis of mtDNA of *P. knowlesi*

The mt genome was selected to examine the evolutionary history of *P. knowlesi*, just as the mt genomes of *P. vivax* [38] and *P. falciparum* [35] were previously found suitable; it does not undergo recombination so intraspecific phylogenetic analysis can be performed and it shows no evidence of non-neutral polymorphism.

DNA sequence data were aligned using the Lasergene package (DNASTAR). Measures of genetic diversity were conducted using DnaSP v5.10.00 software [44]. A minimum spanning network connecting the mtDNA haplotypes of *P. knowlesi* based on statistical parsimony method was constructed using the TCS 1.21 software [45].

Host-parasite association was assessed based on the association index (AI) [21] and parsimony score (PS) statistics [22], which account for phylogenetic uncertainty in analysis of phylogeny-trait correlations. The values of AI and PS statistics were calculated based on the posterior samples of trees produced by BEAST using the BaTS program [44] [46]. The null distribution for each statistic was estimated with 1,000 replicates of state randomization.

The demographic expansion of *P. knowlesi* was examined based on pairwise mismatch distribution using Arlequin v3.1 software [47]. Observed mismatch distribution was compared with that estimated under the sudden demographic expansion model using a generalized least-square approach [48]. The deviations from the population expansion model were tested using the Harpending's raggedness index [23] with a parametric bootstrap of 1000 replicates. Tajima's D [24], Fu and Li's D [25], Fu and Li's F [25] and Fay and Wu's H [26] statistics were performed using the software DnaSP v5.10.00 [44]. These statistics were calculated using the mitochondrial genome of *P. coatneyi* (AB354575) as out-group.

The evolutionary rate, time to the most recent common ancestor (TMRCA) and the past population dynamics of *P. knowlesi* were inferred using the Bayesian Markov Chain Monte Carlo (MCMC) method implemented in the BEAST package v1.5.4 [19]. The mean substitution rate of mtDNA and TMRCA of *P. knowlesi* were estimated based on a time-calibrated Bayesian phylogenetic analysis of non-human primate malarias (*P. gonderi*, *Plasmodium* sp. (Mandrill), *P. simiovale*, *P. fragile*, *P. cynomolgi* and *P. knowlesi*) and human malarias (*P. falciparum*, *P. vivax*, *P. malariae* and *P. ovale*) (Table S6) (Figure 3), assuming co-divergence of the parasites with their host lineages [38], Asian Old World monkeys - African Old World monkeys at 10 MYA [20]. The accession numbers of sequences derived from GenBank database are as follows; *P. falciparum* (M99416), *P. malariae* (AB354570), *P. vivax* (NC007243), *P. ovale* (AB354571), *P. gonderi* (AB434918), *Plasmodium* sp. (mandrill) (AY800112), *P. simiovale* (AB434920), *P. inui* (AB354572), *P. hylobati* (AB354573), *P. cynomolgi* (AB434919), *P. simium* (AY800110), *P. fragile* (AY722799) and *P. coatneyi* (AB354575). A General Time Reversible (GTR) substitution model with gamma distribution of rate variation among sites and a proportion of invariable sites as determined using Modeltest v3.7 [49], an uncorrelated log-normal relaxed molecular clock model and a Bayesian skyline coalescent model (10 coalescent-interval groups) were used for this analysis. One hundred million generations of the MCMC chains were run with sampling every 10,000 generations and the first 10 million generations were discarded as burn-in. The BEAST output was analyzed using the Tracer v1.5 program (available at http://tree.bio.ed.ac.uk/software/tracer/) and uncertainty in parameter estimates was expressed as values of the 95% highest probability density (HPD). The trees produced by BEAST were annotated using TreeAnnotator, and maximum clade credibility tree was visualized using the FigTree v1.3.1 program (available at http://tree.bio.ed.ac.uk/software/figtree/).

Past population dynamics of *P. knowlesi* parasites in terms of the change in effective population size (*Ne*) through time were independently analyzed using the *P. knowlesi* mtDNA datasets for humans and macaque, and also by combining both human and macaque *P. knowlesi* mtDNA datasets. Using the estimated mean substitution rate and Bayesian skyline coalescent model, the MCMC chains were run for 100 million generations with sampling every 10,000 generations and the first 10 million generations were discarded as burn-in.

The changes in effective population size (*N_e_*) through time were also drawn for *M. fascicularis* and *M. nemestrina* based on the cytochrome b sequences obtained from GenBank (Table S7). A BEAST analysis to determine the mean rate substitution of the cytochrome b (*cyt*b) gene of macaques was performed using *cyt*b sequences of *M. fascicularis*, *M. nemestrina* and *Papio anubis* (GenBank accession EU885461), and assuming baboons and macaques diverged 6.6 MYA [50]. An estimated mean substitution rate of 4.56×10^−8^ substitutions per site per year was used to infer the Bayesian skyline plot for *M. fascicularis* and *M. nemestrina*. For each species, 100 millions generations were performed, with sampling every 10,000 generations and 10 percent of the sampling were discard as burn-in.

For all analyses implemented in BEAST, at least 2 independent runs were performed and convergence of all parameters was determined based on Effective Sample Size (ESS) values of >200.

### GenBank accession numbers

The sequences generated during this study have been deposited in GenBank: *P. knowlesi* mitochondrial genome sequence data under the accession numbers EU880446–EU880499 and *P. knowlesi csp* gene sequences under the accession numbers AY327558–AY327572, DQ350272–DQ350306, DQ641526–DQ641528 and GU002471–GU002533.

## Supporting Information

Figure S1 Phylogenetic tree of *Plasmodium* species based on the non-repeat regions of the *csp* genes produced by the neighbor-joining method. Clones derived from macaques have the prefixes LT (long-tailed) or PT (pig-tailed) while those from humans have prefixes KH or CDK. Figures on the branches are bootstrap percentages based on 1,000 replicates and only those above 70% are shown. The horizontal branch length indicates nucleotide substitutions per site computed using the Kimura 2-parameter method. Parasite clones that are underlined represent DNA sequences that are completely identical for the whole *csp* gene. GenBank accession numbers are in brackets and for the sequences with prefixes LT, PTK and CDK that were generated for this study, GenBank accession numbers are provided in Table S1.
(TIF)

Figure S2 Polymorphic sites in the non-repeat regions of *P. knowlesi csp* genes. Clones derived from macaques have prefixes LT or PT while those from humans have prefixes KH or CDK. Clones that are underlined indicate DNA sequences that are completely identical for the whole *csp* gene.
(TIF)

Figure S3 Polymorphisms within the 37 mitochondrial haplotypes of *P. knowlesi* from Kapit Division. Sequences derived from different hosts are indicated as: KH (human), LT (long-tailed macaque) and PT (pig-tailed macaque). Positions of polymorphic sites are numbered vertically on top. Region of gene encoding the cytochrome oxidase subunit I (cox I), cytochrome oxidae subunit III (cox III) and cytochrome b (cyt b) are indicated above the nucleotide positions. Dots represent identical nucleotide residues and dashes represent deletions. Sequence data were deposited in the GenBank database under the accession numbers EU880446–EU880499.
(TIF)

Figure S4 Bayesian skyline plots showing the past population growth through time for *P. knowlesi* isolates derived from humans and macaques. The effective population size (y-axis) is given on a logarithmic scale and time (x-axis) in thousands of years ago. The thick solid black line is the median estimate and the blue shaded area represents the 95% highest probability density (HPD) intervals for effective population size. Both Bayesian skyline plots were estimated using the same model applied to the plots in Figure 4.
(TIF)

Figure S5 Bayesian skyline plots showing the past population growth through time for (A) *Macaca fascicularis* and (B) *Macaca nemestrina*. The effective population size (y-axis) is given on a logarithmic scale. The thick solid black line is the median estimate and the blue shaded area represents the 95% highest probability density (HPD) for effective population size. Note that the effective population size for both hosts declined between 100,000 to 10,000 years before present.
(TIF)

Table S1 Comparison of the repeat motifs of the *csp* genes for *P. knowlesi* isolates derived from human and macaque samples. Each of the different motifs is represented by italicized letters. Clones derived from macaques have prefixes LT (long-tailed) or PT (pig-tailed) while those from humans have prefixes KH or CDK.
(DOC)

Table S2 Tests for recombination of the mitochondrial genome of *P. knowlesi*. (A) “inner fragments” and “outer fragments”, which are evidence of possible gene conversion events resulting from recombination were identified based on comparison between all pairs of sequences in the alignment. *P*-values were calculated by comparing the observed maximum fragment score to the maximum fragment score from permuted data set (10,000 permutations). (B) Correlation between linkage disequilibrium, LD measured as *r* ^2^ and physical distance (d), and correlation between LD measured as |D′| and physical distance (d) were measured based on 1,000 permutations of segregating sites. These null distributions were compared to values observed in unpermuted data and *P*-values were expressed as proportion of correlation between LD (r2 or |D′|) and physical distance that are greater than the observed values.
(DOC)

Table S3 Phylogeny-trait association test of *P. knowlesi*–host clustering based on analysis of *P. knowlesi* mitochondrial DNA haplotypes. Statistics of clustering strength based on Parsimony Score (PS), Association Index (AI) and monophyletic clade (MC) size were computed using BaTS (Bayesian tip-association significance testing) (Parker J, Rambaut A, Pybus OG (2008) Correlating viral phenotypes with phylogeny: accounting for phylogenetic uncertainty. Infect Genet Evol 8: 239–246.). All plausible trees (10% burn in) generated by BEAST analysis were examined and 1,000 replicates of state randomization were performed. *HPD CIs = highest posterior density confidence intervals. ** Significant at *p*<0.01.
(DOC)

Table S4 Sequences and annealing temperatures of species-specific PCR primers used in nested PCR assays. The genus-specific primers, rPLU1 and rPLU5, were used in the primary (nest 1) amplification followed by the species-specific primers in the nest 2 amplifications as described previously (Singh B, Sung LK, Matusop A, Radhakrishnan A, Shamsul SSG, et al. (2004) A large focus of naturally acquired *Plasmodium knowlesi* infections in human beings. Lancet 363: 1017–1024.). The primers are based on the sequences of the small subunit ribosomal RNA genes.
(DOC)

Table S5 Sequences of internal PCR primers used for sequencing mitochondrial DNA of *P. knowlesi*.
(DOC)

Table S6 Mitochondrial DNA sequences of simian malaria parasites used in the estimation of nucleotide substitution rate.
(DOC)

Table S7 Sequences of cytochrome b gene used in the analysis of past population size dynamics of *M. fascicularis* and *M. nemestrina*.
(DOC)

## References

[1] Singh B, Sung LK, Matusop A, Radhakrishnan A, Shamsul SSG (2004). A large focus of naturally acquired *Plasmodium knowlesi* infections in human beings.. Lancet.

[2] Lee KS, Cox-Singh J, Singh B (2009). Morphological features and differential counts of *Plasmodium knowlesi* parasites in naturally acquired human infections.. Malar J.

[3] Cox-Singh J, Davis TME, Lee KS, Shamsul SSG, Matusop A (2008). *Plasmodium knowlesi* malaria in humans is widely distributed and potentially life-threatening.. Clin Infect Dis.

[4] Vythilingam I, NoorAzian YM, Huat TC, Jiram AI, Yusri YM (2008). *Plasmodium knowlesi* in humans, macaques and mosquitoes in peninsular Malaysia.. Parasit Vectors.

[5] Jongwutiwes S, Putaporntip C, Iwasaki T, Sata T, Kanbara H (2004). Naturally acquired *Plasmodium knowlesi* malaria in human, Thailand.. Emerg Infect Dis.

[6] Putaporntip C, Hongsrimuang T, Seethamchai S, Kobasa T, Limkittikul K (2009). Differential prevalence of Plasmodium infections and cryptic *Plasmodium knowlesi* malaria in humans in Thailand.. J Infect Dis.

[7] Zhu HM, Li J, Zheng H (2006). Human natural infection of *Plasmodium knowlesi*.. Chin J Parasitol Parasit Dis.

[8] Ng OT, Ooi EE, Lee CC, Lee PJ, Ng LC (2008). Naturally acquired human *Plasmodium knowlesi* infection, Singapore.. Emerg Infect Dis.

[9] Ong CWM, Lee SY, Koh WH, Ooi EE, Tambyah PA (2009). Monkey malaria in humans: a diagnostic dilemma with conflicting laboratory data.. Am J Trop Med Hyg.

[10] Luchavez J, Espino FE, Curameng P, Espina R, Bell D (2008). Human infections with *Plasmodium knowlesi*, the Philippines.. Emerg Infect Dis.

[11] Eede PV, Van HN, Van Overmeir C, Vythilingam I, Duc TN (2009). Human *Plasmodium knowlesi* infections in young children in central Vietnam.. Malar J.

[12] Figtree M, Lee R, Bain L, Kennedy T, Mackertich S (2010). *Plasmodium knowlesi* in Human, Indonesian Borneo.. Emerg Infect Dis.

[13] Sulistyaningsih E, Fitri LE, Löscher T, Berens-Riha N (2010). Diagnostic difficulties with *Plasmodium knowlesi* infection in humans.. Emerg Infect Dis.

[14] Daneshvar C, Davis TME, Cox-Singh J, Rafa'ee MZ, Zakaria SK (2009). Clinical and laboratory features of human *Plasmodium knowlesi* infection.. Clin Infect Dis.

[15] Knowles R, Das Gupta BM (1932). A study of monkey-malaria and its experimental transmission to man.. Ind Med Gaz.

[16] Garnham PCC (1966). Malaria parasites and other haemosporidia.

[17] Lambrecht FL, Dunn FL, Eyles DE (1961). Isolation of *Plasmodium knowlesi* from Philippine macaques.. Nature.

[18] Chin W, Contacos PG, Collins WE, Jeter MH, Alpert E (1968). Experimental mosquito-transmission of *Plasmodium knowlesi* to man and monkey.. Am J Trop Med Hyg.

[19] Drummond AJ, Rambaut A, Shapiro B, Pybus OG (2005). Bayesian coalescent inference of past population dynamics from molecular sequences.. Mol Biol Evol.

[20] Hayakawa T, Culleton R, Otani H, Horii T, Tanabe K (2008). Big bang in the evolution of extant malaria parasites.. Mol Biol Evol.

[21] Wang TH, Donaldson YK, Brettle RP, Bell JE, Simmonds P (2001). Identification of shared populations of human immunodeficiency virus type 1 infecting microglia and tissue macrophages outside the central nervous system.. J Virol.

[22] Slatkin M, Maddison WP (1989). A cladistic measure of gene flow inferred from the phylogenies of alleles.. Genetics.

[23] Harpending HC (1994). Signature of ancient population growth in a low-resolution mitochondrial DNA mismatch distribution.. Hum Biol.

[24] Tajima F (1989). Statistical method for testing the neutral mutation hypothesis by DNA polymorphism.. Genetics.

[25] Fu YX, Li WH (1993). Statistical tests of neutrality of mutations.. Genetics.

[26] Fay JC, Wu CI (2000). Hitchhiking under positive Darwinian selection.. Genetics.

[27] Aris-Brosou S, Excoffier L (1996). The impact of population expansion and mutation rate heterogeneity on DNA sequence polymorphism.. Mol Biol Evol.

[28] Coatney GR, Collins WE, Warren M, Contacos PG (1971). The primate malarias.

[29] Seethamchai S, Putaporntip C, Malaivijitnond S, Cui L, Jongwutiwes S (2008). Malaria and Hepatocystis species in wild macaques, southern Thailand.. Am J Trop Med Hyg.

[30] Vythilingam I, Tan CH, Asmad M, Chan ST, Lee KS (2006). Natural transmission of *Plasmodium knowlesi* to humans by Anopheles latens in Sarawak, Malaysia.. Trans R Soc Trop Med Hyg.

[31] Tan CH, Vythilingam I, Matusop A, Chan ST, Singh B (2008). Bionomics of *Anopheles latens* in Kapit, Sarawak, Malaysian Borneo in relation to the transmission of zoonotic simian malaria parasite *Plasmodium knowlesi*.. Malar J.

[32] Macaulay V, Hill C, Achilli A, Rengo C, Clarke D (2005). Single, rapid coastal settlement of Asia revealed by analysis of complete mitochondrial genomes.. Science.

[33] Soares P, Ermini L, Thomson N, Mormina M, Rito T (2009). Correcting for purifying selection: an improved human mitochondrial molecular clock.. Am J Hum Genet.

[34] Ziegler T, Abegg C, Meijaard E, Perwitasari-Farajallah D, Walter L (2007). Molecular phylogeny and evolutionary history of Southeast Asian macaques forming the *M. silenus* group.. Mol Phylogenet Evol.

[35] Joy DA, Feng X, Mu J, Furuya T, Chotivanich K (2003). Early origin and recent expansion of *Plasmodium falciparum*.. Science.

[36] Krief S, Escalante AA, Pacheco MA, Mugisha L, André C (2010). On the diversity of malaria parasites in African apes and the origin of *Plasmodium falciparum* from Bonobos.. PLoS Pathog.

[37] Escalante AA, Cornejo OE, Freeland DE, Poe AC, Durrego E (2005). A monkey's tale: the origin of *Plasmodium vivax* as a human malaria parasite.. Proc Natl Acad Sci U S A.

[38] Mu J, Joy DA, Duan J, Huang Y, Carlton J (2005). Host switch leads to emergence of *Plasmodium vivax* malaria in humans.. Mol Biol Evol.

[39] Voris HK (2000). Maps of Pleistocene sea levels in Southeast Asia: shorelines, river systems and time durations.. J Biogeog.

[40] Atkinson QD, Gray RD, Drummond AJ (2008). mtDNA variation predicts population size in humans and reveals a major Southern Asian chapter in human prehistory.. Mol Biol Evol.

[41] Pain A, Böhme U, Berry AE, Mungall K, Finn RD (2008). The genome of the simian and human malaria parasite *Plasmodium knowlesi*.. Nature.

[42] Liu W, Li Y, Learn GH, Rudicell RS, Robertson JD (2010). Origin of the human malaria parasite *Plasmodium falciparum* in gorillas.. Nature.

[43] Jongwutiwes S, Putaporntip C, Iwasaki T, Ferreira MU, Kanbara H (2005). Mitochondrial genome sequences support ancient population expansion in *Plasmodium vivax*.. Mol Biol Evol.

[44] Librado P, Rozas J (2009). DnaSP v5: a software for comprehensive analysis of DNA polymorphism data.. Bioinformatics.

[45] Clement M, Posada D, Crandall KA (2000). TCS: a computer program to estimate gene genealogies.. Mol Ecol.

[46] Parker J, Rambaut A, Pybus OG (2008). Correlating viral phenotypes with phylogeny: accounting for phylogenetic uncertainty.. Infect Genet Evol.

[47] Excoffier L, Laval G, Schneider S (2005). Arlequin (version 3.0): an integrated software package for population genetics data analysis.. Evol Bioinform Online.

[48] Schneider S, Excoffier L (1999). Estimation of past demographic parameters from the distribution of pairwise differences when the mutation rates vary among sites: application to human mitochondrial DNA.. Genetics.

[49] Posada D, Crandall KA (1998). MODELTEST: testing the model of DNA substitution.. Bioinformatics.

[50] Steiper ME, Young NM (2006). Primate molecular divergence dates.. Mol Phylogenet Evol.

