# Supplementary figures and images for: Plasmodium knowlesi: Reservoir Hosts and Tracking the Emergence in Humans and Macaques
Source: PLoS Pathog. 2011 Apr 7;7(4):e1002015. doi: 10.1371/journal.ppat.1002015 (PMC3072369; doi:10.1371/journal.ppat.1002015)

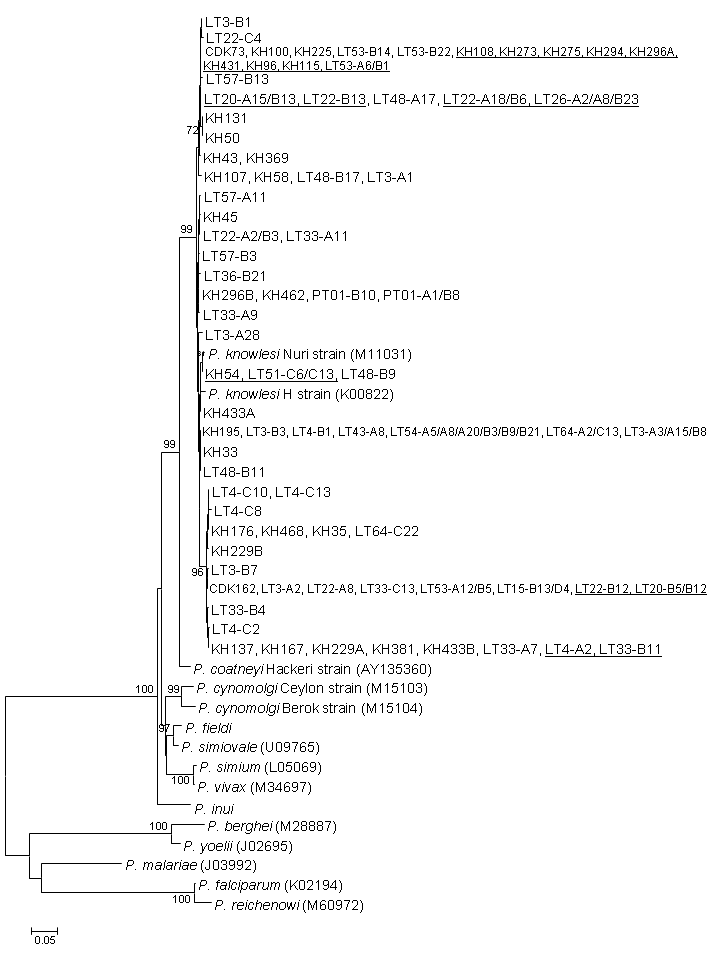

Supplement: Figure S1 — Phylogenetic tree of Plasmodium species based on the non-repeat regions of the csp genes produced by the neighbor-joining method. Clones derived from macaques have the prefixes LT (long-tailed) or PT (pig-tailed) while those from humans have prefixes KH or CDK. Figures on the branches are bootstrap percentages based on 1,000 replicates and only those above 70% are shown. The horizontal branch length indicates nucleotide substitutions per site computed using the Kimura 2-parameter method. Parasite clones that are underlined represent DNA sequences that are completely identical for the whole csp gene. GenBank accession numbers are in brackets and for the sequences with prefixes LT, PTK and CDK that were generated for this study, GenBank accession numbers are provided in Table S1. (TIF) [file ppat.1002015.s001.tif]

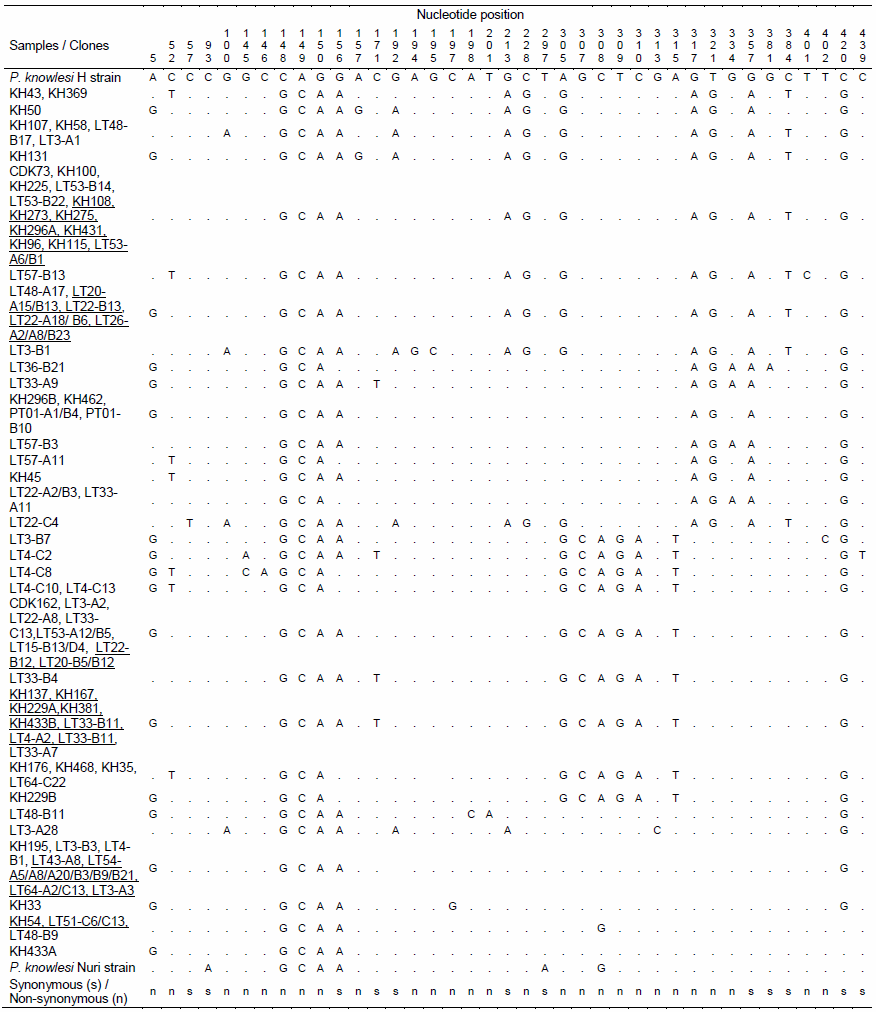

Supplement: Figure S2 — Polymorphic sites in the non-repeat regions of P. knowlesi csp genes. Clones derived from macaques have prefixes LT or PT while those from humans have prefixes KH or CDK. Clones that are underlined indicate DNA sequences that are completely identical for the whole csp gene. (TIF) [file ppat.1002015.s002.tif]

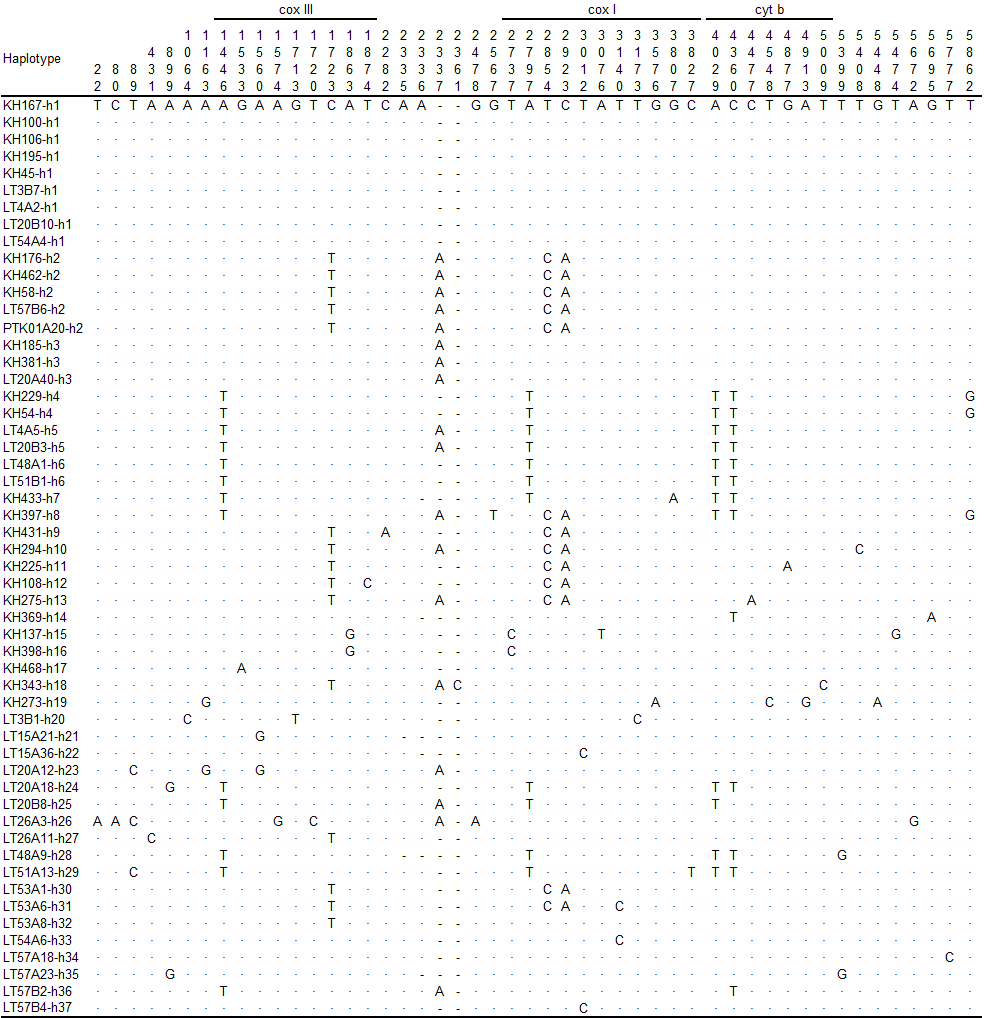

Supplement: Figure S3 — Polymorphisms within the 37 mitochondrial haplotypes of P. knowlesi from Kapit Division. Sequences derived from different hosts are indicated as: KH (human), LT (long-tailed macaque) and PT (pig-tailed macaque). Positions of polymorphic sites are numbered vertically on top. Region of gene encoding the cytochrome oxidase subunit I (cox I), cytochrome oxidae subunit III (cox III) and cytochrome b (cyt b) are indicated above the nucleotide positions. Dots represent identical nucleotide residues and dashes represent deletions. Sequence data were deposited in the GenBank database under the accession numbers EU880446–EU880499. (TIF) [file ppat.1002015.s003.tif]

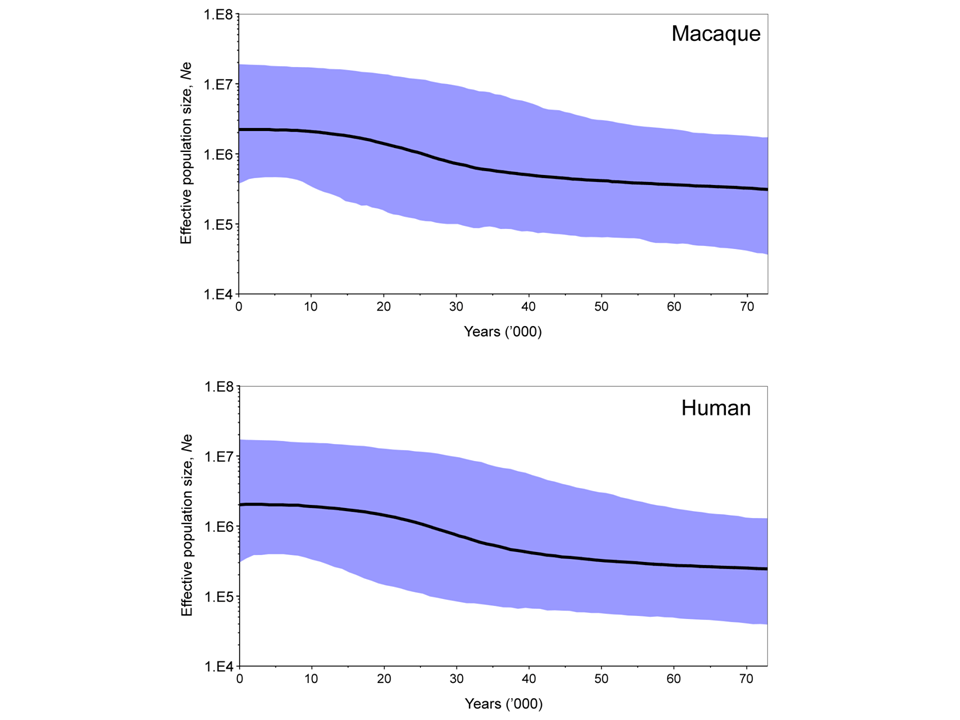

Supplement: Figure S4 — Bayesian skyline plots showing the past population growth through time for P. knowlesi isolates derived from humans and macaques. The effective population size (y-axis) is given on a logarithmic scale and time (x-axis) in thousands of years ago. The thick solid black line is the median estimate and the blue shaded area represents the 95% highest probability density (HPD) intervals for effective population size. Both Bayesian skyline plots were estimated using the same model applied to the plots in Figure 4. (TIF) [file ppat.1002015.s004.tif]

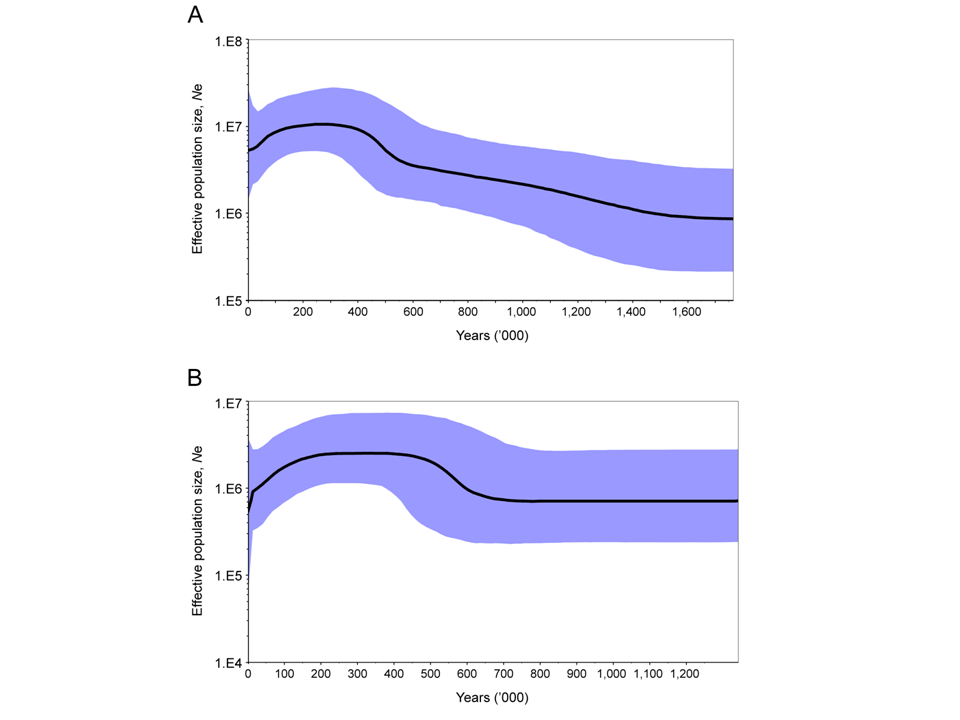

Supplement: Figure S5 — Bayesian skyline plots showing the past population growth through time for (A) Macaca fascicularis and (B) Macaca nemestrina. The effective population size (y-axis) is given on a logarithmic scale. The thick solid black line is the median estimate and the blue shaded area represents the 95% highest probability density (HPD) for effective population size. Note that the effective population size for both hosts declined between 100,000 to 10,000 years before present. (TIF) [file ppat.1002015.s005.tif]
