# Supplementary material for: Plasmodium knowlesi: Reservoir Hosts and Tracking the Emergence in Humans and Macaques
Source: PLoS Pathog. 2011 Apr 7;7(4):e1002015. doi: 10.1371/journal.ppat.1002015 (PMC3072369; doi:10.1371/journal.ppat.1002015)
Supplement: Table S1 — Comparison of the repeat motifs of the csp genes for P. knowlesi isolates derived from human and macaque samples. Each of the different motifs is represented by italicized letters. Clones derived from macaques have prefixes LT (long-tailed) or PT (pig-tailed) while those from humans have prefixes KH or CDK. (DOC) [file ppat.1002015.s006.doc]

**Table S1.** Comparison of the repeat motifs of the *csp* genes for *P. knowlesi* isolates derived from human and macaque samples.

| Sample  (GenBank accession number) | Total size of *csp* (bp) | Repeat motifs (a.a.) |  | No. of each repeat motif | Sequence of repeat motifs | Size of tandem repeat region (bp) |
| --- | --- | --- | --- | --- | --- | --- |
| H strain (K00822) | 1092 | NEGQPQAQGDGA | *A* | 1 | *ABBBBBBBBBBB* | 432 |
|  |  | NAGQPQAQGDGA | *B* | 11 |  |  |
| LT4-B1 (DQ350294) | 1113 | NPGQPQAQGDGA | *j* | 1 | *jBBBBBBBBBBNB* | 468 |
|  |  | NAGQPQAQGDGA | *B* | 11 |  |  |
|  |  | NAGQPQAQGDRA | *N* | 1 |  |  |
| LT48-B11 (GU002510) | 1074 | NPDQPQAQGDGA | *d* | 1 | *dBBBBBBBBBJ* | 396 |
|  |  | NAGQPQAQGDGA | *B* | 9 |  |  |
|  |  | NAGQPQAQGDRG | *J* | 1 |  |  |
| KH195 (GU002479) | 1077 | NPDQPQAQGDGA | *d* | 1 | *dBBBBBBBBBNB* | 432 |
|  |  | NAGQPQAQGDGA | *B* | 10 |  |  |
|  |  | NAGQPQAQGDRA | *N* | 1 |  |  |
| KH33 (AY327558) | 1080 | NEGQPQAQGDGA | *A* | 1 | *ABBBBBBBBBNB* | 432 |
|  |  | NAGQPQAQGDGA | *B* | 10 |  |  |
|  |  | NAGQPQAQGDRA | *N* | 1 |  |  |
| CDK162 (GU002486) | 1077 | NPDQPQAQGDGA | *d* | 1 | *dBBBBBBBBBNB* | 432 |
|  |  | NAGQPQAQGDGA | *B* | 10 |  |  |
|  |  | NAGQPQAQGDRA | *N* | 1 |  |  |
| KH433A (GU002480) | 1113 | NPGQPQAQGDGA | *j* | 1 | *jBBBBBBBBBBNB* | 468 |
|  |  | NAGQPQAQGDGA | *B* | 11 |  |  |
|  |  | NAGQPQAQGDRA | *N* | 1 |  |  |
| LT3-A3/A15 (DQ350276, DQ350280), LT43-A8 (GU002507), LT54-A5/A8/A20/B3/B9/B21 (GU002508), LT64-A2/C13 (GU002509) | 1077 | NPDQPQAQGDGA | *d* | 1 | *dBBBBBBBBBNB* | 432 |
|  |  | NAGQPQAQGDGA | *B* | 10 |  |  |
|  |  | NAGQPQAQGDRA | *N* | 1 |  |  |
| Nuri strain (M11031) | 1053 | EQPAAGAGG | *C* | 11 | *CCCDCCCCCDCCDCE* | 405 |
|  |  | EQPAAGARG | *D* | 3 |  |  |
|  |  | EQPAPAPRR | *E* | 1 |  |  |
| LT48-B9 (GU002506) | 1026 | EQPAAGAGG | *C* | 11 | *CCCCCCCCDCCECE* | 378 |
|  |  | EQPAAGARG | *D* | 1 |  |  |
|  |  | EQPAPAPRR | *E* | 2 |  |  |
| KH54 (GU002491), LT51-C6/C13 (GU002531) | 1053 | EQPAAGAGG | *C* | 11 | *CCCCCCDCCDCCECE* | 405 |
|  |  | EQPAAGARG | *D* | 2 |  |  |
|  |  | EQPAPAPRR | *E* | 2 |  |  |
| LT48-A17 (GU002496) | 999 | NAEGGANAGQP | *F* | 10 | *FFFFFFFFFFGH* | 399 |
|  |  | NAEGGANARQP | *G* | 1 |  |  |
|  |  | QAEGGGANARQG | *H* | 1 |  |  |
| KH107 (AY327568) | 1095 | NAEGGANAGQP | *F* | 11 | *FFFFFFFGFFGFFGH* | 498 |
|  |  | NAEGGANARQP | *G* | 3 |  |  |
|  |  | QAEGGGANARQG | *H* | 1 |  |  |
| LT22-A18/B6 (GU002497), LT26-A2/A8/B23 (GU002530) | 1098 | NAEGGANAGQP | *F* | 12 | *FFFFFFFGFFFFFGH* | 498 |
|  |  | NAEGGANARQP | *G* | 2 |  |  |
|  |  | QAEGGGANARQG | *H* | 1 |  |  |
| LT20-A15/B13 (GU002495), LT22-B13 (GU002529) | 1098 | NAEGGANAGQP | *F* | 12 | *FFFFFFFFFFGFFGH* | 498 |
|  |  | NAEGGANARQP | *G* | 2 |  |  |
|  |  | QAEGGGANARQG | *H* | 1 |  |  |
| LT53-B14 (GU002492) | 1038 | NAEGDGGNARQP | *O* | 7 | *OOOOOOOPQ* | 324 |
|  |  | NAEGDGANARQP | *P* | 1 |  |  |
|  |  | QAEGGGGNARQG | *Q* | 1 |  |  |
| KH100 (GU002488) | 1218 | NAEGDGGNARQP | *O* | 11 | *OOOOOOOOOOOPPQ* | 504 |
|  |  | NAEGDGANARQP | *P* | 2 |  |  |
|  |  | QAEGGGGNARQG | *Q* | 1 |  |  |
| KH225 (GU002489) | 1182 | NAEGDGGNARQP | *O* | 12 | *OOOOOOOOOOOOPQ* | 504 |
|  |  | NAEGDGANARQP | *P* | 1 |  |  |
|  |  | QAEGGGGNARQG | *Q* | 1 |  |  |
| CDK73 (GU002487) | 1218 | NAEGDGGNARQP | *O* | 12 | *OOOOOOOOOOOOPQ* | 504 |
|  |  | NAEGDGANARQP | *P* | 1 |  |  |
|  |  | QAEGGGGNARQG | *Q* | 1 |  |  |
| KH96 (AY327566), KH108 (GU002490), KH115 (AY327570), KH273 (GU002524), KH275 (GU002525), KH294 (GU002533), KH296A (GU002526), KH431 (GU002527), LT53-A6/B1 (GU002528) | 1218 | NAEGDGGNARQP | *O* | 12 | *OOOOOOOOOOOOPQ* | 504 |
|  |  | NAEGDGANARQP | *P* | 1 |  |  |
|  |  | QAEGGGGNARQG | *Q* | 1 |  |  |
| LT53-B22 (GU002493) | 714 | NAEGDGANARQP | *P* | 1 | *PQ* | 72 |
|  |  | QAEGGGGNARQG | *Q* | 1 |  |  |
| KH58 (GU002484) | 1101 | NAEGDGANARQP | *P* | 11 | *PPPPPPPPPPoPY* | 468 |
|  |  | NAEGGGANARQP | *o* | 1 |  |  |
|  |  | QAQGDGGNARQG | *Y* | 1 |  |  |
| LT57-A11 (GU002499) | 1077 | EGNQDGRAQP | *T* | 9 | *TTTTTTTTTUVTVUV* | 459 |
|  |  | EGNREAPAQP | *U* | 2 |  |  |
|  |  | QGNGGAGQAQP | *V* | 3 |  |  |
| KH43 (AY327562) | 1068 | EGNQDGRAQP | *T* | 9 | *TTTTTTTTTUVUVUV* | 459 |
|  |  | EGNREAPAQP | *U* | 3 |  |  |
|  |  | QGNGGAGQAQP | *V* | 3 |  |  |
| KH45 (GU002483) | 1077 | EGNQDGRAQP | *T* | 9 | *TTTTTTTTTUVUVUV* | 459 |
|  |  | EGNREAPAQP | *U* | 3 |  |  |
|  |  | QGNGGAGQAQP | *V* | 3 |  |  |
| LT57-B13 (GU002494) | 1068 | EGNQDGRAQP | *T* | 9 | *TTTTTTTTTUVUVUV* | 459 |
|  |  | EGNREAPAQP | *U* | 3 |  |  |
|  |  | QGNGGAGQAQP | *V* | 3 |  |  |
| LT3-A1 (DQ350272) | 1092 | QAEGDGANARQP | *W* | 10 | *WWWWWWyWWWXWY* | 468 |
|  |  | QAEGDGANARQS | *y* | 1 |  |  |
|  |  | QAEGGGANARQP | *X* | 1 |  |  |
|  |  | QAQGDGGNARQG | *Y* | 1 |  |  |
| LT3-B1 (DQ350284) | 1092 | QAEGDGANARQP | *W* | 11 | *WWWWWWWWWWXWY* | 468 |
|  |  | QAEGGGANARQP | *X* | 1 |  |  |
|  |  | QAQGDGGNARQG | *Y* | 1 |  |  |
| LT3-A28 (DQ350282) | 1092 | QAEGDGANARQP | *W* | 11 | *WWWWWWWWWWXWY* | 468 |
|  |  | QAEGGGANARQP | *X* | 1 |  |  |
|  |  | QAQGDGGNARQG | *Y* | 1 |  |  |
| LT22-C4 (DQ641528) | 1092 | QAEGDGANARQP | *W* | 11 | *WWWWWWWWWWXWY* | 468 |
|  |  | QAEGGGANARQP | *X* | 1 |  |  |
|  |  | QAQGDGGNARQG | *Y* | 1 |  |  |
| LT48-B17 (GU002498) | 1092 | QAEGDGANARQP | *W* | 11 | *WWWWWWWWWWXWY* | 468 |
|  |  | QAEGGGANARQP | *X* | 1 |  |  |
|  |  | QAQGDGGNARQG | *Y* | 1 |  |  |
| KH50 (AY327564) | 1092 | QAEGDGANARQP | *W* | 10 | *WWWWWWWWWXXWY* | 468 |
|  |  | QAEGGGANARQP | *X* | 2 |  |  |
|  |  | QAQGDGGNARQG | *Y* | 1 |  |  |
| KH131 (AY327572) | 1092 | QVEGDGANARQP | *w* | 1 | *wWWWWWWWWXXWY* | 468 |
|  |  | QAEGDGANARQP | *W* | 9 |  |  |
|  |  | QAEGGGANARQP | *X* | 2 |  |  |
|  |  | QAQGDGGNARQG | *Y* | 1 |  |  |
| KH369 (GU002485) | 1092 | QAEGDGANARQP | *W* | 11 | *WWWWWWWWWWXWY* | 468 |
|  |  | QAEGGGANARQP | *X* | 1 |  |  |
|  |  | QAQGDGGNARQG | *Y* | 1 |  |  |
| LT22-A2 (DQ641526) | 1035 | EQARPGG | *k* | 1 | *keeeeeeeememeEen* | 426 |
|  |  | EQPAPGPGG | *e* | 11 |  |  |
|  |  | EQPAPRPGG | *m* | 2 |  |  |
|  |  | EQPAPAPRR | *E* | 1 |  |  |
|  |  | EQPAPGAGA | *n* | 1 |  |  |
| LT22-B3 (DQ641527) | 1035 | EQARPGG | *k* | 1 | *keeeeeeeememeEen* | 426 |
|  |  | EQPAPGPGG | *e* | 11 |  |  |
|  |  | EQPAPRPGG | *m* | 2 |  |  |
|  |  | EQPAPAPRR | *E* | 1 |  |  |
|  |  | EQPAPGAGA | *n* | 1 |  |  |
| LT33-C13 (GU002514) | 1011 | GGEQPAA | *R* | 11 | *RRRRSRRRRSRRSRKLRM* | 390 |
|  |  | GGERPAA | *S* | 3 |  |  |
|  |  | GGEQPAP | *K* | 2 |  |  |
|  |  | APRREQPAA | *L* | 1 |  |  |
|  |  | APRREQPAP | *M* | 1 |  |  |
| LT36-B21 (GU002502) | 990 | GGEQPAA | *R* | 12 | *RRRRRRRRRRRKLRKMK* | 369 |
|  |  | GGEQPAP | *K* | 3 |  |  |
|  |  | APRREQPAA | *L* | 1 |  |  |
|  |  | APRREQPAP | *M* | 1 |  |  |
| LT53-A12/B5 (GU002515) | 1014 | EQERPGG | *i* | 1 | *ieemeeemememeEf* | 399 |
|  |  | EQPAPGPGG | *e* | 8 |  |  |
|  |  | EQPAPRPGG | *m* | 4 |  |  |
|  |  | EQPAPAPRR | *E* | 1 |  |  |
|  |  | EQPAPAPGA | *f* | 1 |  |  |
| LT64-C22 (GU002511) | 1011 | GGEQPAA | *R* | 11 | *RRRRRRSRRSRSRRKLKM* | 390 |
|  |  | GGERPAA | *S* | 3 |  |  |
|  |  | GGEQPAP | *K* | 2 |  |  |
|  |  | APRREQPAA | *L* | 1 |  |  |
|  |  | APRREQPAP | *M* | 1 |  |  |
| KH35 (AY327560) | 1032 | GGEQPAA | *R* | 12 | *RRRRRRRSRRSRSRRKLKL* | 411 |
|  |  | GGERPAA | *S* | 3 |  |  |
|  |  | GGEQPAP | *K* | 2 |  |  |
|  |  | APRREQPAA | *L* | 2 |  |  |
| LT57-B3 (GU002501) | 1017 | EQARAGG | *g* | 1 | *gppppppppqpppqqpEpn* | 411 |
|  |  | EQPAAGG | *p* | 13 |  |  |
|  |  | ERPAAGG | *q* | 3 |  |  |
|  |  | EQPAPAPRR | *E* | 1 |  |  |
|  |  | EQPAPGAGA | *n* | 1 |  |  |
| LT33-A11 (GU002500) | 966 | EQARAGG | *g* | 1 | *gppppppppppEpEpn* | 354 |
|  |  | EQPAAGG | *p* | 12 |  |  |
|  |  | EQPAPAPRR | *E* | 2 |  |  |
|  |  | EQPAPGAGA | *n* | 1 |  |  |
| KH176 (GU002477) | 990 | GGEQPAA | *R* | 10 | *RRRRRSRRSRSRRKLKM* | 369 |
|  |  | GGERPAA | *S* | 3 |  |  |
|  |  | GGEQPAP | *K* | 2 |  |  |
|  |  | APRREQPAA | *L* | 1 |  |  |
|  |  | APRREQPAP | *M* | 1 |  |  |
| KH229B (GU002476) | 1011 | GGEQPAA | *R* | 11 | *RRRRSRRRRSRRRSKLKM* | 390 |
|  |  | GGERPAA | *S* | 3 |  |  |
|  |  | GGEQPAP | *K* | 2 |  |  |
|  |  | APRREQPAA | *L* | 1 |  |  |
|  |  | APRREQPAP | *M* | 1 |  |  |
| KH468 (GU002478) | 1011 | GGEQPAA | *R* | 11 | *RRRRSRRRRSRRSRKLKM* | 390 |
|  |  | GGERPAA | *S* | 3 |  |  |
|  |  | GGEQPAP | *K* | 2 |  |  |
|  |  | APRREQPAA | *L* | 1 |  |  |
|  |  | APRREQPAP | *M* | 1 |  |  |
| PT01-A1/B8 (GU002504) | 657 | EQAAPRR | *r* | 1 | *rf* | 48 |
|  |  | EQPAPAPGA | *f* | 1 |  |  |
| LT22-A8 (GU002513) | 1089 | DGAGPGG | *s* | 1 | *sttutututtutututumEvn* | 480 |
|  |  | EQAGPGG | *t* | 9 |  |  |
|  |  | EQAGPRPGG | *u* | 7 |  |  |
|  |  | EQPAPRPGG | *m* | 1 |  |  |
|  |  | EQPAPAPRR | *E* | 1 |  |  |
|  |  | EQPAPAPGG | *v* | 1 |  |  |
|  |  | EQPAPGAGA | *n* | 1 |  |  |
| LT4-C8 (DQ350302) | 1068 | DGAGPGG | *s* | 1 | *sttututututututuEen* | 459 |
|  |  | EQAGPGG | *t* | 8 |  |  |
|  |  | EQAGPRPGG | *u* | 7 |  |  |
|  |  | EQPAPAPRR | *E* | 1 |  |  |
|  |  | EQPAPGPGG | *e* | 1 |  |  |
|  |  | EQPAPGAGA | *n* | 1 |  |  |
| LT3-A2 (DQ350274) | 1041 | EQAAPGAGG | *Z* | 1 | *ZaabaabaaaabbaEc* | 432 |
|  |  | EQPAPGAGG | *a* | 9 |  |  |
|  |  | ERPAPGAGG | *b* | 4 |  |  |
|  |  | EQPAPAPRR | *E* | 1 |  |  |
|  |  | EQPAPGPGA | *c* | 1 |  |  |
| LT3-B3 (DQ350286) | 1041 | EQAAPGAGG | *Z* | 1 | *ZabaaaabaaababEc* | 432 |
|  |  | EQPAPGAGG | *a* | 9 |  |  |
|  |  | ERPAPGAGG | *b* | 4 |  |  |
|  |  | EQPAPAPRR | *E* | 1 |  |  |
|  |  | EQPAPGPGA | *c* | 1 |  |  |
| LT3-B7 (GU002512) | 1038 | EQAAPGAGG | *Z* | 1 | *ZaabaabaaaahbaEc* | 432 |
|  |  | EQPAPGAGG | *a* | 9 |  |  |
|  |  | ERPAPGAGG | *b* | 3 |  |  |
|  |  | ELPAPGAGG | *h* | 1 |  |  |
|  |  | EQPAPAPRR | *E* | 1 |  |  |
|  |  | EQPAPGPGA | *c* | 1 |  |  |
| LT4-C2 (DQ350300) | 1041 | EQAAPGAGG | *Z* | 1 | *ZabaaaabaaababEc* | 432 |
|  |  | EQPAPGAGG | *a* | 9 |  |  |
|  |  | ERPAPGAGG | *b* | 4 |  |  |
|  |  | EQPAPAPRR | *E* | 1 |  |  |
|  |  | EQPAPGPGA | *c* | 1 |  |  |
| LT4-C10 (DQ350304) | 1068 | DQAAPGAGG | *z* | 1 | *zaabaabbaaaabbaEc* | 459 |
|  |  | EQPAPGAGG | *a* | 9 |  |  |
|  |  | ERPAPGAGG | *b* | 4 |  |  |
|  |  | EQPAPAPRR | *E* | 1 |  |  |
|  |  | EQPAPGPGA | *c* | 1 |  |  |
| LT4-C13 (DQ350306) | 1041 | DQAAPGAGG | *z* | 1 | *zaabaabaaaabbaEc* | 432 |
|  |  | EQPAPGAGG | *a* | 9 |  |  |
|  |  | ERPAPGAGG | *b* | 4 |  |  |
|  |  | EQPAPAPRR | *E* | 1 |  |  |
|  |  | EQPAPGPGA | *c* | 1 |  |  |
| LT33-B4 (GU002518) | 1041 | EQAAPGAGG | *Z* | 1 | *ZabaaaabaaababEc* | 432 |
|  |  | EQPAPGAGG | *a* | 9 |  |  |
|  |  | ERPAPGAGG | *b* | 4 |  |  |
|  |  | EQPAPAPRR | *E* | 1 |  |  |
|  |  | EQPAPGPGA | *c* | 1 |  |  |
| KH462 (GU002482) | 1041 | EQAAPGAGG | *Z* | 1 | *ZabaaaabababaaEf* | 432 |
|  |  | EQPAPGAGG | *a* | 9 |  |  |
|  |  | ERPAPGAGG | *b* | 4 |  |  |
|  |  | EQPAPAPRR | *E* | 1 |  |  |
|  |  | EQPAPAPGA | *f* | 1 |  |  |
| LT15-B13/D4 (GU002516) | 1068 | EQAAPGAGG | *Z* | 1 | *ZaaabaabaaaabbaEc* | 459 |
|  |  | EQPAPGAGG | *a* | 10 |  |  |
|  |  | ERPAPGAGG | *b* | 4 |  |  |
|  |  | EQPAPAPRR | *E* | 1 |  |  |
|  |  | EQPAPGPGA | *c* | 1 |  |  |
| LT22-B12 (GU002517),  LT20-B5/B12 (GU002532) | 1041 | EQAAPGAGG | *Z* | 1 | *ZaabaabaaaabbaEc* | 432 |
|  |  | EQPAPGAGG | *a* | 9 |  |  |
|  |  | ERPAPGAGG | *b* | 4 |  |  |
|  |  | EQPAPAPRR | *E* | 1 |  |  |
|  |  | EQPAPGPGA | *c* | 1 |  |  |
| KH137 (GU002471), KH167 (GU002472), KH229A (GU002473), KH381 (GU002474), KH433B (GU002475), LT33-B11 (GU002520), LT4-A2 (DQ350292) | 1041 | EQAAPGAGG | *Z* | 1 | *ZabaaaabaaababEc* | 432 |
|  |  | EQPAPGAGG | *a* | 9 |  |  |
|  |  | ERPAPGAGG | *b* | 4 |  |  |
|  |  | EQPAPAPRR | *E* | 1 |  |  |
|  |  | EQPAPGPGA | *c* | 1 |  |  |
| LT33-A9 (GU002505) | 936 | EQAAPGAGG | *Z* | 1 | *ZabaaappEpEpn* | 327 |
|  |  | EQPAPGAGG | *a* | 4 |  |  |
|  |  | ERPAPGAGG | *b* | 1 |  |  |
|  |  | EQPAAGG | *p* | 4 |  |  |
|  |  | EQPAPAPRR | *E* | 2 |  |  |
|  |  | EQPAPGAGA | *n* | 1 |  |  |
| LT33-A7 (GU002519) | 798 | EQAAPGAGG | *Z* | 1 | *ZababEc* | 189 |
|  |  | EQPAPGAGG | *a* | 2 |  |  |
|  |  | ERPAPGAGG | *b* | 2 |  |  |
|  |  | EQPAPAPRR | *E* | 1 |  |  |
|  |  | EQPAPGPGA | *c* | 1 |  |  |
| KH296B (GU002481) | 798 | EQAAPGAGG | *Z* | 1 | *ZaaaaEf* | 189 |
|  |  | EQPAPGAGG | *a* | 4 |  |  |
|  |  | EQPAPAPRR | *E* | 1 |  |  |
|  |  | EQPAPAPGA | *f* | 1 |  |  |
| PT01-B10 (GU002503) | 717 | EQAAPGAGG | *Z* | 1 | *ZaEf* | 108 |
|  |  | EQPAPGAGG | *a* | 1 |  |  |
|  |  | EQPAPAPRR | *E* | 1 |  |  |
|  |  | EQPAPAPGA | *f* | 1 |  |  |
|  |  |  |  |  |  |  |

Each of the different motifs is represented by italicised letters. Clones derived from macaques have prefixes LT (long-tailed) or PT (pig-tailed) while those from humans have prefixes KH or CDK.
