# Supplementary material for: Plasmodium knowlesi: Reservoir Hosts and Tracking the Emergence in Humans and Macaques
Source: PLoS Pathog. 2011 Apr 7;7(4):e1002015. doi: 10.1371/journal.ppat.1002015 (PMC3072369; doi:10.1371/journal.ppat.1002015)
Supplement: Table S2 — Tests for recombination of the mitochondrial genome of P. knowlesi. (A) “inner fragments” and “outer fragments”, which are evidence of possible gene conversion events resulting from recombination were identified based on comparison between all pairs of sequences in the alignment. P-values were calculated by comparing the observed maximum fragment score to the maximum fragment score from permuted data set (10,000 permutations). (B) Correlation between linkage disequilibrium, LD measured as r 2 and physical distance (d), and correlation between LD measured as |D′| and physical distance (d) were measured based on 1,000 permutations of segregating sites. These null distributions were compared to values observed in unpermuted data and P-values were expressed as proportion of correlation between LD (r2 or |D′|) and physical distance that are greater than the observed values. (DOC) [file ppat.1002015.s007.doc]

**Table S2.** Tests for recombination of the mitochondrial genome of *P. knowlesi.*

A. GENECONV

|  | Maximum Fragment Score | *P* |
| --- | --- | --- |
| Inner fragments | 3.335 | 0.1288 |
| Outer fragments | 0.385 | 0.6988 |

**B. LDhat**

|  | Correlation coefficient | *P* | |
| --- | --- | --- | --- |
| *r*^2^ and d | -0.03043 | 0.2340 |  |
| |D’| and d | 0.00502 | 0.5390 |  |

**(A)** “inner fragments” and “outer fragments”, which are evidence of possible gene conversion events resulting from recombination were identified based on comparison between all pairs of sequences in the alignment. *P*-values were calculated by comparing the observed maximum fragment score to the maximum fragment score from permuted data set (10,000 permutations). **(B)** Correlation between linkage disequilibrium, LD measured as *r*^2^ and physical distance (d), and correlation between LD measured as |D’| and physical distance (d) were measured based on 1,000 permutations of segregating sites. These null distributions were compared to values observed in unpermuted data and *P*-values were expressed as proportion of correlation between LD (r2 or |D’|) and physical distance that are greater than the observed values.
