# Supplementary material for: Plasmodium knowlesi: Reservoir Hosts and Tracking the Emergence in Humans and Macaques
Source: PLoS Pathog. 2011 Apr 7;7(4):e1002015. doi: 10.1371/journal.ppat.1002015 (PMC3072369; doi:10.1371/journal.ppat.1002015)
Supplement: Table S3 — Phylogeny-trait association test of P. knowlesi–host clustering based on analysis of P. knowlesi mitochondrial DNA haplotypes. Statistics of clustering strength based on Parsimony Score (PS), Association Index (AI) and monophyletic clade (MC) size were computed using BaTS (Bayesian tip-association significance testing) (Parker J, Rambaut A, Pybus OG (2008) Correlating viral phenotypes with phylogeny: accounting for phylogenetic uncertainty. Infect Genet Evol 8: 239–246.). All plausible trees (10% burn in) generated by BEAST analysis were examined and 1,000 replicates of state randomization were performed. *HPD CIs = highest posterior density confidence intervals. ** Significant at p<0.01. (DOC) [file ppat.1002015.s008.doc]

**Table S3.** Phylogeny-trait association test of *P. knowlesi*–host clustering based on analysis of *P. knowlesi* mitochondrial DNA haplotypes.

| **Statistic** | **BaTS estimate (95% *HPD CIs)** | *****p*-value** |
| --- | --- | --- |
| **AI** | 2.35 (1.50, 3.20) | 0.012 |
| **PS** | 16.10 (13, 18) | 0.044 |
| **MC (human)** | 3.25 (2, 5) | 0.340 |
| **MC (macaque)** | 4.13 (2, 7) | 0.067 |

Statistics of clustering strength based on Parsimony Score (PS), Association Index (AI) and monophyletic clade (MC) size were computed using BaTS (Bayesian tip-association significance testing) (Parker et al., 2008). All plausible trees (10% burn in) generated by BEAST analysis were examined and 1,000 replicates of state randomization were performed. *HPD CIs = highest posterior density confidence intervals. ** Significant at *p*<0.01.

Parker J, Rambaut A, Pybus OG (2008) Correlating viral phenotypes with phylogeny: accounting for phylogenetic uncertainty. Infect Genet Evol 8: 239-246.
