# Supplementary material for: Plasmodium knowlesi: Reservoir Hosts and Tracking the Emergence in Humans and Macaques
Source: PLoS Pathog. 2011 Apr 7;7(4):e1002015. doi: 10.1371/journal.ppat.1002015 (PMC3072369; doi:10.1371/journal.ppat.1002015)
Supplement: Table S4 — Sequences and annealing temperatures of species-specific PCR primers used in nested PCR assays. The genus-specific primers, rPLU1 and rPLU5, were used in the primary (nest 1) amplification followed by the species-specific primers in the nest 2 amplifications as described previously (Singh B, Sung LK, Matusop A, Radhakrishnan A, Shamsul SSG, et al. (2004) A large focus of naturally acquired Plasmodium knowlesi infections in human beings. Lancet 363: 1017–1024.). The primers are based on the sequences of the small subunit ribosomal RNA genes. (DOC) [file ppat.1002015.s009.doc]

**Table S4.** Sequences and annealing temperatures of species-specific PCR primers used in nested PCR assays

| *Plasmodium* | Primers | Sequence (5’ – 3’) | Annealing temperature (^o^C) |
| --- | --- | --- | --- |
| *knowlesi* | Kn1f | CTCAACACGGGAAAACTCACTAGTTTA | 62 |
|  | Kn3r | GTATTATTAGGTACAAGGTAGCAGTATGC |  |
| *coatneyi* | PctF1 | CGCTTTTAGCTTAAATCCACATAACAGAC | 62 |
|  | PctR1 | GAGTCCTAACCCCGAAGGGAAAGG |  |
| *cynomolgi* | CY2F | GATTTGCTAAATTGCGGTCG | 60 |
|  | CY4R | CGGTATGATAAGCCAGGGAAGT |  |
| *inui* | PinF2 | CGTATCGACTTTGTGGCATTTTTCTAC | 60 |
|  | INAR3 | gcaatctaagagttttaactcctc |  |
| *fieldi* | PfldF1 | GGTCTTTTTTTTGCTTCGGTAATTA | 66 |
|  | PfldR2 | AGGCACTGAAGGAAGCAATCTAAGAGTTTC |  |

The genus-specific primers, rPLU1 and rPLU5, were used in the primary (nest 1) amplification followed by the species-specific primers in the nest 2 amplifications as described previously (Singh et al., 2004). The primers are based on the sequences of the small subunit ribosomal RNA genes.

Singh B, Sung LK, Matusop A, Radhakrishnan A, Shamsul SSG, et al. (2004) A large focus of naturally acquired *Plasmodium knowlesi* infections in human beings. Lancet 363: 1017-1024.
