# Supplementary material for: Plasmodium knowlesi: Reservoir Hosts and Tracking the Emergence in Humans and Macaques
Source: PLoS Pathog. 2011 Apr 7;7(4):e1002015. doi: 10.1371/journal.ppat.1002015 (PMC3072369; doi:10.1371/journal.ppat.1002015)
Supplement: Table S5 — Sequences of internal PCR primers used for sequencing mitochondrial DNA of P. knowlesi. (DOC) [file ppat.1002015.s010.doc]

**Table S5.** Sequences of internal PCR primers used for sequencing mitochondrial DNA of *P. knowlesi.*

| Primer | Sequence (5’ – 3’) |
| --- | --- |
| *Forward* |  |
| Pkmt-seq1F | TCCACACTTCAATTCGTACTTCCA |
| Pkmt-seq2F | CGTCGGGCCGTATGATTCCACA |
| Pkmt-seq3F | GCTCACGCATCGCTTCTAACG |
| Pkmt-seq4F | AGACGCTGACTTCCTGGCTAAAC |
| Pkmt-seq5F | CACTTCCCTTCTCGCCATTTGATA |
| Pkmt-seq6F | TGCTTTATTATGGATTGGATGTCA |
| Pkmt-seq7F | TGCAAAACATTCTCCAAGTAAGT |
| Pkmt-seq8F | ATGATACAGCTTTAAAAATACCCTTCT |
| Pkmt-seq9F | TTCGCCGGGGATAACAGGT |
| Pkmt-seq10F | AAGAGAATTATGGAGTGGATGGTG |
| Pkmt-seq11F | CGAACGCTTTTAACGCCTGAC |
| Pkmt-seq12F | TGCTCATTTTCACTTTGTATTATCTAT |
| Pkmt-seq13F | ATCACTTATGTCTTTATCTCCTGTTGC |
| Pkmt-seq14F | ACATCTATTCTGGTTCTTTGGACATC |
|  |  |
| *Reverse* |  |
| Pkmt-seq1R | TGGAAGTACGAATTGAAGTGTGGA |
| Pkmt-seq2R | TTCCCCATTGTCGCTAGTGTGAGA |
| Pkmt-seq3R | ACCGTTAGAAGCGATGCGTGAGC |
| Pkmt-seq4R | ACACCAGGCATGCAATACCGAACA |
| Pkmt-seq5R | TATCAAATGGCGAGAAGGGAAGTG |
| Pkmt-seq6R | TTGACATCCAATCCATAATAAAGC |
| Pkmt-seq7R | AATATACTTACTTGGAGAATG |
| Pkmt-seq8R | TAGAAGGGTATTTTTAAAGCTGTATCA |
| Pkmt-seq9R | CAAGGCTGCGATGAGACGACA |
| Pkmt-seq10R | TATATCTAAAACACCATCCACTCCAT |
| Pkmt-seq11R | GGCGTTAAAAGCGTTCGTTCT |
| Pkmt-seq12R | TCCAAGAAAATGCATAGGTAAGAA |
| Pkmt-seq13R | TGCAACAGGAGATAAAGACATAAGTGA |
| Pkmt-seq14R | TGAGCCCATACAACACTTCCTA |
