# Supplementary material for: Plasmodium knowlesi: Reservoir Hosts and Tracking the Emergence in Humans and Macaques
Source: PLoS Pathog. 2011 Apr 7;7(4):e1002015. doi: 10.1371/journal.ppat.1002015 (PMC3072369; doi:10.1371/journal.ppat.1002015)
Supplement: Table S6 — Mitochondrial DNA sequences of simian malaria parasites used in the estimation of nucleotide substitution rate. (DOC) [file ppat.1002015.s011.doc]

**Table S6.** Mitochondrial DNA sequences of simian malaria parasites used in the estimation of nucleotide substitution rate.

| **Species** | **Natural host** | **GenBank accession number** |
| --- | --- | --- |
| *Plasmodium gonderi* | Mangabeys, drills | AY800111 |
| *Plasmodium simiovale* | *Macaca sinica* | AY800109 |
| *Plasmodium cynomolgi* | *M. fascicularis*  *M. nemestrina*  *M. radiata*  *M. sinica*  *M. cyclopis*  *Presbytis cristatus*  *P. entellus* | AY800108 |
| *Plasmodium fragile* | *M. radiata*  *M. sinica* | AY722799 |
