# Supplementary material for: Plasmodium knowlesi: Reservoir Hosts and Tracking the Emergence in Humans and Macaques
Source: PLoS Pathog. 2011 Apr 7;7(4):e1002015. doi: 10.1371/journal.ppat.1002015 (PMC3072369; doi:10.1371/journal.ppat.1002015)
Supplement: Table S7 — Sequences of cytochrome b gene used in the analysis of past population size dynamics of M. fascicularis and M. nemestrina. (DOC) [file ppat.1002015.s012.doc]

**Table S7.** Sequences of cytochrome b gene used in the analysis of past population size dynamics of *M. fascicularis* and *M. nemestrina*.

| Host | GenBank accession number |
| --- | --- |
| *Macaca fascicularis* | DQ373460, DQ373465, DQ373468, DQ373483, DQ373497, DQ373507, DQ373510, DQ373511, DQ373526, DQ373599, DQ373604, DQ373612, DQ373617, DQ373624, DQ373656, DQ373657, DQ373661, DQ373667, DQ373675, DQ373679, DQ373685, DQ373691, DQ373699, DQ373701, DQ373710, DQ373711, DQ373724, DQ373730, DQ373736, DQ373744, DQ373751, DQ373753, DQ373757, DQ373769, DQ373784, DQ373786, DQ373806, DQ373814, DQ373825, DQ373829, DQ373843, DQ373852, DQ373856, DQ373857, DQ373858, DQ373860, DQ373875, DQ373885, DQ373893, DQ373897 |
| *Macaca nemestrina* | EU204975, AF350397, AF350390, AF350391, AF350388, AF350389AF350396, AF350395, AF350394, AF350398, AF350399, DQ355483, DQ355484, DQ355486, AY151097, AY151102, AY151104, AY151103, AY151110, DQ355485, AY151109, AY151107, AY151106, AY151113, AY151112, AY151111, AY151105, AY151108, AY151094, AY151101, AY151099, AY151092, AY151091, AY151098, AY151096, AY151093, AY151100, AY151095 |
